# A Physiologically Informed Strategy to Effectively Open, Stabilize, and Protect the Acutely Injured Lung

**DOI:** 10.3389/fphys.2020.00227

**Published:** 2020-03-19

**Authors:** Gary F. Nieman, Hassan Al-Khalisy, Michaela Kollisch-Singule, Joshua Satalin, Sarah Blair, Girish Trikha, Penny Andrews, Maria Madden, Louis A. Gatto, Nader M. Habashi

**Affiliations:** ^1^Department of Surgery, SUNY Upstate Medical University, Syracuse, NY, United States; ^2^Department of Medicine, SUNY Upstate Medical University, Syracuse, NY, United States; ^3^Department of Pediatric Surgery, Arkansas Children’s Hospital, Little Rock, AR, United States; ^4^Department of Trauma Critical Care Medicine, R Adams Cowley Shock Trauma Center, University of Maryland School of Medicine, Baltimore, MD, United States; ^5^Department of Biological Sciences, SUNY Cortland, Cortland, NY, United States

**Keywords:** ARDS, VILI (ventilator induced lung injury), mechanical ventilalion, APRV, alveolar mechanics

## Abstract

Acute respiratory distress syndrome (ARDS) causes a heterogeneous lung injury and remains a serious medical problem, with one of the only treatments being supportive care in the form of mechanical ventilation. It is very difficult, however, to mechanically ventilate the heterogeneously damaged lung without causing secondary ventilator-induced lung injury (VILI). The acutely injured lung becomes *time* and *pressure* dependent, meaning that it takes more time and pressure to open the lung, and it recollapses more quickly and at higher pressure. Current protective ventilation strategies, ARDSnet low tidal volume (LVt) and the open lung approach (OLA), have been unsuccessful at further reducing ARDS mortality. We postulate that this is because the LVt strategy is constrained to ventilating a lung with a heterogeneous mix of normal and focalized injured tissue, and the OLA, although designed to fully open and stabilize the lung, is often unsuccessful at doing so. In this review we analyzed the pathophysiology of ARDS that renders the lung susceptible to VILI. We also analyzed the alterations in alveolar and alveolar duct mechanics that occur in the acutely injured lung and discussed how these alterations are a key mechanism driving VILI. Our analysis suggests that the *time* component of each mechanical breath, at both inspiration and expiration, is critical to normalize alveolar mechanics and protect the lung from VILI. Animal studies and a meta-analysis have suggested that the time-controlled adaptive ventilation (TCAV) method, using the airway pressure release ventilation mode, eliminates the constraints of ventilating a lung with heterogeneous injury, since it is highly effective at opening and stabilizing the *time-* and *pressure*-dependent lung. In animal studies it has been shown that by “casting open” the acutely injured lung with TCAV we can (1) reestablish normal expiratory lung volume as assessed by direct observation of subpleural alveoli; (2) return normal parenchymal microanatomical structural support, known as alveolar interdependence and parenchymal tethering, as assessed by morphometric analysis of lung histology; (3) facilitate regeneration of normal surfactant function measured as increases in surfactant proteins A and B; and (4) significantly increase lung compliance, which reduces the pathologic impact of driving pressure and mechanical power at any given tidal volume.

## Introduction

**FIGURE 1:**
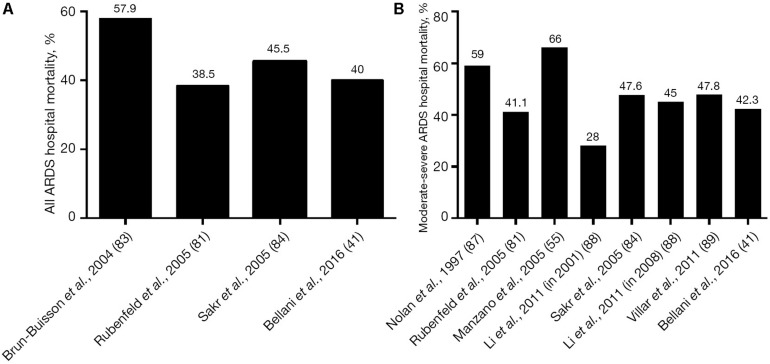
**(A)** Hospital mortality (%) in the main epidemiological studies for all three American-European Consensus Conference (AECC) classifications of acute respiratory distress syndrome (ARDS – mild, moderate, and severe) and **(B)** mortality (%) for only moderate and severe ARDS. The mean and standard deviation for mortality in all of the studies following the 2000 ARMA study (Acute Respiratory Distress Syndrome Network, 2000) is 45.4 ± 9.5 (Rezoagli et al., 2017). *Permissions obtained from AME Publishing Company, License ID 1017423-1.*

Acute respiratory distress syndrome (ARDS) was initially thought to be a lethal double pneumonia and was identified as a syndrome by Ashbaugh et al. (1967). Unfortunately, in the 50 years since ARDS was identified, only a few treatments have been used, with the mainstay being supportive in the form of mechanical ventilation (Slutsky and Ranieri, 2013). However, mechanical ventilation constrained to the limitations of a heterogeneously injured lung can cause unintended tissue damage, referred to as ventilator-induced lung injury (VILI), which can significantly increase mortality (∼40%) as compared to lung protective ventilation (∼31%) (Acute Respiratory Distress Syndrome Network, 2000). Initial randomized controlled trials (RCTs) attempting to reduce VILI by lowering tidal volume (Vt) failed to reduce mortality (Stewart et al., 1998; Brower et al., 1999). It was not until the ARDS Network (ARDSnet) conducted the seminal ARMA study, published in 2000, that a reduction in mortality was shown (Acute Respiratory Distress Syndrome Network, 2000). However, most (Phua et al., 2009; Villar et al., 2011; Caser et al., 2014; Bellani et al., 2016; Laffey et al., 2016; Villar et al., 2016; Maca et al., 2017; Raymondos et al., 2017; Rezoagli et al., 2017; Fan et al., 2018; McNicholas et al., 2018; Pham et al., 2019; Shen et al., 2019) but not all (Brun-Buisson et al., 2004; Fan et al., 2005; Putensen et al., 2009; Petrucci and De Feo, 2013; Shen et al., 2019) of the recent statistical- and meta-analyses have shown that ARDS mortality has not been reduced below the 31% “gold standard” of the 2000 ARMA study but rather remains unacceptably high at ∼40% (Figure 1). Despite these disappointing results, the low-Vt ARDSnet method is still recommended as the standard-of-care protective ventilation strategy for ARDS patients (Fan et al., 2017, 2018; Papazian et al., 2019).

Since outcome data for ARDS patients has not improved for almost 20 years, it is imperative to (1) ascertain the mechanisms of dynamic alveolar and alveolar duct volume change during mechanical ventilation (elastic, viscous, or viscoelastic), (2) characterize ARDS-induced changes in alveolar mechanics (i.e., the dynamic change in alveolar size and shape during mechanical ventilation) (Grune et al., 2019) that drive VILI-induced tissue damage, (3) identify the role of airway pressure and the duration at both inspiration and expiration on alveolar mechanics in the acutely injured lung (Kollisch-Singule et al., 2014a, 2018), and (4) use this knowledge to develop novel ventilation strategies to better reduce VILI and protect the lung. Although pulmonary inflammation (biotrauma) also plays a critical role in ARDS and VILI pathogenesis, the focus of this review will be the mechanical injury to tissue caused during ventilation.

## Constraints of Ventilating the Acutely Injured Lung

**FIGURE 2:**
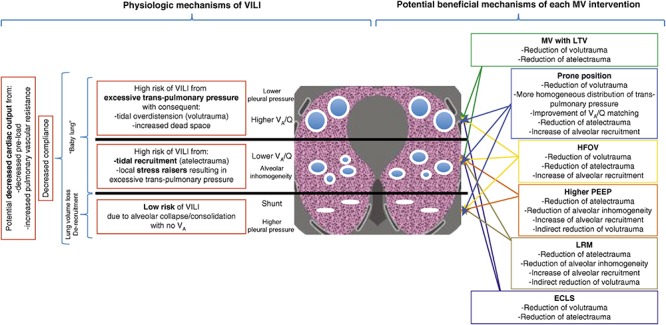
Gravity dependent three-compartment model of acute respiratory distress syndrome (ARDS) pathology: **Left** – Physiological mechanisms of ventilator-induced lung injury (VILI) and **Right** – Potential beneficial mechanisms of various protective ventilation strategies to minimize VILI. Open alveoli are shown as blue circles similar in size (top), unstable alveoli as smaller circles of various sizes (middle), and collapsed alveoli as solid white lines (bottom). The ARDSnet low tidal volume (LVt) method is designed to *protect* compliant open alveoli in the non-dependent lung and *rest* the collapsed tissue in the dependent portion of the lung by keeping it unventilated. Positive end-expiratory pressure (PEEP) is added in an attempt to *stabilize* the alveoli in between (Acute Respiratory Distress Syndrome Network, 2000). High-frequency oscillatory ventilation (HFOV) and lung recruitment maneuvers (LRMs) have been shown ineffective in reducing ARDS mortality (Brower et al., 2004; Meade et al., 2008; Mercat et al., 2008; Ferguson et al., 2013; Young et al., 2013; Cavalcanti et al., 2017; Hodgson et al., 2019). Prone position has been shown effective at reducing mortality (Guerin et al., 2013) by a mechanism of reducing regional alveolar strain and inflammation (Motta-Ribeiro et al., 2018; Xin et al., 2018). VILI, ventilator-induced lung injury; MV, mechanical ventilation; LTV, low tidal volume and inspiratory pressure; PEEP, positive end-expiratory pressure; ECLS, extracorporeal life support; HFOV, high-frequency oscillatory ventilation; LRM, lung recruitment maneuver; Q, perfusion; V_A_, alveolar ventilation; V_A_/Q, ventilation/perfusion ratio; baby lung, functional residual capacity (FRC) (Del Sorbo et al., 2017). *Permissions to publish obtained from ATS.*

The current concept is that ARDS causes heterogeneous injury in three general lung compartments that are layered by gravity (Figure 2). The first compartment, or the non-dependent area, contains a small number of normal compliant alveoli that remain inflated at end-expiration—functional residual capacity (FRC)—and is referred to as the “baby lung” (Gattinoni and Pesenti, 2005). The second compartment consists of alveoli in the dependent areas that are collapsed and/or edema filled. The third compartment consists of alveoli that remain in the transition zone between healthy and unstable, due to loss of surfactant function, and that open and collapse with every breath.

Consequently, the ARDSnet low-Vt and plateau pressure (Pplat) strategy is constrained to ventilating this heterogeneous lung tissue without causing VILI using a three-tiered approach: (1) *protect* the baby lung by not overdistending the compliant tissue that is open at FRC, (2) *rest* the dependent collapsed and edema-filled tissue by keeping it out of the ventilatory cycle, and (3) *stabilize* the tissue in between by applying positive end-expiratory pressure (PEEP), usually adjusted by oxygenation (Figure 2) (Acute Respiratory Distress Syndrome Network, 2000; Del Sorbo et al., 2017).

### Problems With Protecting the Baby Lung

Since the baby lung is believed to be a small volume of normal tissue (Gattinoni and Pesenti, 2005), its overdistension has been postulated to be a primary VILI mechanism (Brower et al., 2004). However, in studies in which normal lungs were subjected to excessively high airway pressures (>30 cm H_2_O) and strain (2.5 ratio), overdistension-induced VILI did not occur as long as this excessive strain was nearly static. This suggests that overdistension of normal tissue with high volume and pressure is not a primary VILI mechanism unless there is also a large dynamic strain (Seah et al., 2011; Protti et al., 2013b; Jain et al., 2017). It is possible that the baby lung is injured not by overdistension of the normal tissue, but rather by the recruiting and recollapse of unstable tissue in the adjacent collapsed regions (Gattinoni et al., 1995). Thus, lowering Vt and Pplat to reduce overdistension in the open lung tissue, which is surrounded by a large volume of collapsed and unstable tissue of very low compliance, may not reduce VILI. Regional instability and inflammation occur throughout the entire lung, including in tissue that appears to be normal on computed tomography (CT) scan or chest X-ray, and serve as pathologic focal points from which VILI-induced tissue damage expands (Wellman et al., 2014, 2016; Cereda et al., 2016b, 2017). This suggests that to protect the normal lung tissue regional instability must be eliminated.

However, others have shown that overdistension is a major component of VILI pathophysiology in injured lungs (Guldner et al., 2016). Guldner et al. (2016), in a porcine ARDS model, showed that extreme conditions of overdistension resulted in more lung inflammation than did extreme lung collapse, suggesting that static stress and strain are major VILI mechanisms. Although this study clearly showed that volutrauma increased inflammation, histopathology was not measured, and there was no difference in pulmonary edema as measured by lung weight. Thus, it is not clear whether the increase in inflammation caused any lung pathology. In addition, recalculation of the data showed that the average mechanical power in the volutrauma group was 17.12 J/min, more than double that of the atelectrauma group (7.13 J/min) (Tonetti et al., 2017). Others have also shown that increasing airway pressure in an acutely injured lung will cause a rapid progression of injury in a “rich-get-richer” power-law fashion, supporting the findings in the Guldner study (Hamlington et al., 2018). Combined, these studies suggest that high static stress and strain are associated with volutrauma in acutely injured lung tissue but not in normal lung tissue.

### Problems With Resting the Collapsed Lung

When the lung is allowed to collapse below normal FRC, atelectatic, and edema-filled *resting* tissue (1) does not exchange gas; (2) is susceptible to the development of pneumonia (Huynh et al., 2019; Li Bassi et al., 2019); (3) will become fibrotic if not reopened (Burkhardt, 1989; Cabrera-Benitez et al., 2014; Lutz et al., 2015); (4) initiates patient-ventilator dyssynchrony, which is caused by the firing of mechanical stretch, PO_2_, PCO_2_, and pH receptors (Solomon et al., 2000; Manning and Mahler, 2001; Widdicombe, 2001; Mellott et al., 2009; Burki and Lee, 2010; Yu, 2016; Yoshida et al., 2017) and which is associated with high mortality (Blanch et al., 2015); and (5) creates a stress-focus in the adjacent open alveoli and alveolar ducts, greatly amplifying the forces applied to these parenchymal tissues during tidal ventilation (Mead et al., 1970; Gattinoni et al., 2012; Cressoni et al., 2014; Makiyama et al., 2014; Retamal et al., 2014). It has been shown that letting the lung *rest* in obese bariatric surgery patients is associated with worse oxygenation, longer post-anesthesia care unit stay, and more post-operative pulmonary complications as compared with patients in which the atelectatic *resting* lung was opened and ventilated (Talab et al., 2009). Although, the phrase *resting* the lung sounds protective, the lung is not meant to function in a deflated state, and as listed above, such a state is associated with numerous pathologies. If the lung is to be *rested*, meaning that parenchymal tissue is to be kept from being damaged by mechanical ventilation, the better strategy would be to *rest* it in the natural inflated state (Nieman et al., 2018).

### Problems With Stabilizing the Lung

There is no consensus on how best to set PEEP to effectively *stabilize* lung tissue (Coruh and Luks, 2014; Gattinoni et al., 2017; Nieman et al., 2017a; Bergez et al., 2019). The current ARDSnet method for setting PEEP uses a sliding scale of oxygenation (Acute Respiratory Distress Syndrome Network, 2000), but increased oxygenation does not correlate well with an increase in alveolar stability (Andrews et al., 2015), a key VILI mechanism (Wellman et al., 2014, 2016; Cereda et al., 2016b, 2017). Many methods have been used in an attempt to titrate the PEEP to stabilize lung tissue. These methods include using dead space, lung stress and strain, lung compliance, CT, pressure-volume curve inflection points, and electrical impedance tomography, but there is no current bedside technique to determine whether the set PEEP has actually stabilized the lung (Nieman et al., 2017a). The above problems with the ARDSnet *protect*, *rest*, and *stabilize* method may partially explain the lack of improved outcome in ARDS mortality over the last 20 years (Figure 1) (Brun-Buisson et al., 2004; Phua et al., 2009; Villar et al., 2011; Caser et al., 2014; Bellani et al., 2016; Laffey et al., 2016; Villar et al., 2016; Maca et al., 2017; Raymondos et al., 2017; Rezoagli et al., 2017; Fan et al., 2018; McNicholas et al., 2018; Pham et al., 2019). By allowing the lung to remain heterogeneously collapsed, the *protect*, *rest*, and *stabilize* method is unintendedly preserving the constraints of ventilating the heterogeneously injured lung, which is nearly impossible to do without causing some degree of VILI.

### Open Lung Approach (OLA) as a Protective Strategy

The goal of the open lung approach (OLA) is to eliminate the constraints of ventilating a heterogeneously injured lung by normalizing all three pathologic compartments (Figure 2). The aim is to reinflate the collapsed tissue using a recruitment maneuver (RM) and to keep it open by using an appropriate level of PEEP. If the entire lung could be recruited and recollapse prevented, the main VILI mechanical mechanisms (dynamic strain and overdistension of alveolar walls in areas of stress-focus) would be eliminated (Nieman et al., 2017b). An RM is an acute event performed by raising the airway pressure (30–40 cm H_2_O) and holding it for ∼40 s (Fan et al., 2008) or by greatly increasing PEEP (25 cm H_2_O) and combining it with 15 cm H_2_O of driving pressure above the PEEP (Borges et al., 2006). In the latter strategy, PEEP is increased in 5 cm H_2_O increments up to 45 cm H_2_O until the lung fully recruits, which was confirmed when PaO_2_ + PaCO_2_ > 400 mmHg (Borges et al., 2006).

Following the RM, PEEP is titrated downward to find the lung recollapse point (usually by a sharp fall in lung compliance), and then PEEP is set 2 cm H_2_O above this collapse pressure, following a second RM. However, multiple RCTs testing the OLA in ARDS patients have failed to show significant benefits over standard of care (Brower et al., 2004; Meade et al., 2008; Mercat et al., 2008; Cavalcanti et al., 2017; Hodgson et al., 2019). Reasons for these failures include the following: (1) timing of OLA application [early (Borges et al., 2006) vs. late (Gattinoni et al., 2006)] (2) one-size-fits-all RM strategies, (3) PEEP set inappropriately to keep the recruited lung open, (4) recruiting pressures insufficient to open all of the lung, (5) a patient population of responders (lung recruits) and non-responders (lung does not recruit) (Gattinoni et al., 2006), and (6) application of OLA not as a continuous treatment but rather as a one-time event with a long time period before a second application or with no second application at all (Goligher et al., 2017; Lu et al., 2017; Bhattacharjee et al., 2018; Cui et al., 2019; Hodgson et al., 2019; Kang et al., 2019; van der Zee and Gommers, 2019; Zheng et al., 2019). Most (Bhattacharjee et al., 2018; Cui et al., 2019; Hodgson et al., 2019; Kang et al., 2019; Zheng et al., 2019) but not all (Goligher et al., 2017; Lu et al., 2017) meta-analyses have shown no decrease in ARDS-related mortality associated with the OLA.

To further reduce ARDS mortality and acute lung injury, two pathologic processes must be understood: (1) the pathophysiology of ARDS that predisposes the lung to a secondary VILI and (2) the mechanisms of VILI in the microenvironment (i.e., the terminal airspaces, alveoli, and alveolar ducts). This knowledge informs the design of a protective mechanical breath that will allow the lung to heal by eliminating the constraints present when ventilating a heterogeneously injured lung (Nieman et al., 2018).

## ARDS Pathophysiology That Predisposes the Lung to VILI

### ARDS Is a Pathologic Tetrad

**FIGURE 3:**
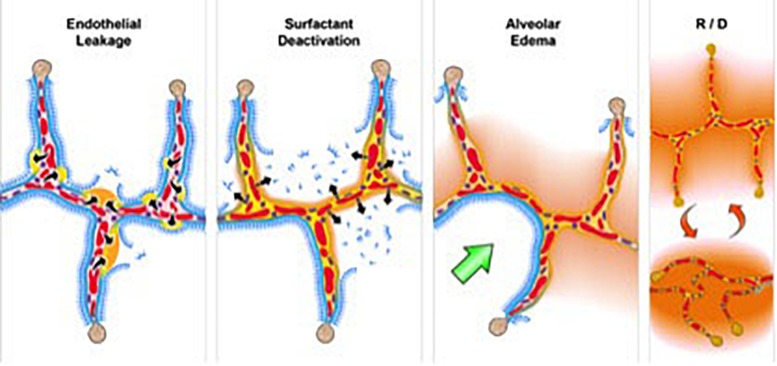
The *pathologic tetrad* of acute respiratory distress syndrome (ARDS). Alveolar walls contain pulmonary capillaries (red circles) and are lined with a liquid hypophase (blue layer inside each alveolus), with an intact pulmonary surfactant layer (small blue ball with tail) layered on the hypophase. The systemic inflammatory response syndrome (SIRS) secondary to sepsis, trauma, burns, pneumonia, and so on increases pulmonary capillary permeability. **Endothelial leakage:** increased microvascular permeability allowing pulmonary edema to move into the alveolus (black arrows and tan edema blebs) (Martin and Brigham, 2012). **Surfactant deactivation:** the continuous layer of pulmonary surfactant molecules is disrupted as the edema blebs expand causing surfactant deactivation (surfactant sluffing off into the alveolar space). Edema usurping surfactant from the alveolar surface, the proteins in the edema fluid deactivating the surfactant (Taeusch et al., 2005), and improper mechanical ventilation (Albert, 2012) causing further surfactant disruption all combine to exacerbate surfactant loss. **Alveolar edema:** increased capillary permeability (Martin and Brigham, 2012) and high alveolar surface tension combine to flood alveoli with edema fluid (tan). **Recruitment**/**derecruitment** (**R**/**D**): loss of surfactant function results in increased alveolar surface tension causing loss of alveolar stability (i.e., causing alveolar R/D with each breath). Alveoli in the top frame of **R/D** are fully inflated but collapse during expiration in the bottom **R/D** frame. Alveolar R/D, known as atelectrauma, is another key VILI mechanism (Cressoni et al., 2017). **Stress-focus**: edema-filled or collapsed alveoli adjacent to air-filled alveoli create a stress-focus causing the alveolar wall to bend toward the fluid-filled alveolus (green arrow), which can cause stress failure at the alveolar wall (Perlman et al., 2011). Stress-focus is another key mechanism of VILI (Perlman et al., 2011; Chen et al., 2014; Makiyama et al., 2014; Retamal et al., 2014). Thus, the *pathologic tetrad* sets up a vicious cycle of high microvascular permeability → edema → surfactant deactivation → high alveolar surface tension → more edema → alveolar R/D → further increase in microvascular permeability → severe ARDS (Nieman and Bredenberg, 1985).

To investigate the relationship between ARDS and VILI, we need to understand the pathology of acute lung injury. Although ARDS is a complex syndrome, it features four well-accepted central components (Thompson et al., 2017) known as the “pathologic tetrad” (Figure 3) (Nieman et al., 2018). The components of the tetrad include *increased pulmonary capillary permeability* (Figure 3, Endothelial Leakage), which if unchecked will lead to *loss of surfactant function* (Figure 3, Surfactant Deactivation) (Lewis et al., 1993). The resultant high alveolar surface tension will exacerbate the permeability-induced increase in *alveolar flooding* with edema fluid (Figure 3, Alveolar Edema) (Nieman and Bredenberg, 1985). Surfactant dysfunction will alter alveolar mechanics, resulting in alveolar *recruitment/derecruitment (R/D)* with each breath (Figure 3, R/D) (Schiller et al., 2001). Each component of the tetrad has a profound impact on alveolar mechanics. Surfactant deactivation sets the stage for a secondary VILI by promoting heterogeneous lung tissue instability and collapse. In addition, surfactant secretions from type II cells would be inhibited in collapsed areas that are not being stretched during ventilation (Wirtz and Dobbs, 1990; Majumdar et al., 2012). Reduced surfactant secretion would exacerbate and perpetuate the already reduced surfactant function caused by alveolar flooding with edema.

## VILI Mechanisms: Heterogeneous Alveolar Instability and Collapse

The hallmark of ARDS is a heterogeneous lung injury encompassing normal, collapsed, edematous, and unstable tissues (Figure 2). This pathology alters pulmonary microanatomy and dynamic alveolar inflation physiology, generating three basic VILI mechanisms: *volutrauma* (overdistension of airways), *atelectrauma* (R/D of alveoli), and *biotrauma* (inflammation) (Thompson et al., 2017). From an engineering perspective, volutrauma is caused by excessive static strain and atelectrauma by excessive dynamic strain (Seah et al., 2011; Protti et al., 2013a, b, 2014). In this review we do not discuss biotrauma but rather focus on the unintentional mechanical damage to the pulmonary parenchyma caused during mechanical ventilation.

**FIGURE 4:**
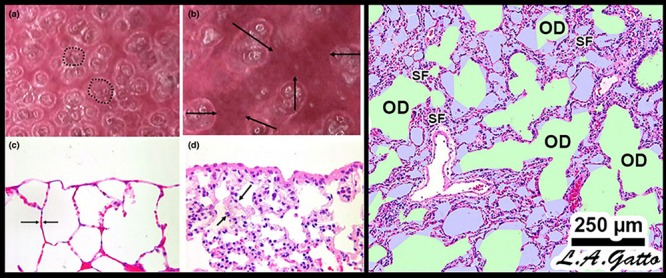
Mechanical mechanisms of ventilator-induced lung injury (VILI) in the microenvironment: Left – *In vivo* subpleural alveoli in an acutely injured rat lung at inspiration **(a)** and expiration **(b)**. Lung histology in the same rat lung injury model fixed at inspiration **(c)** and expiration **(d)** (Pavone et al., 2007). *Alveolar recruitment/derecruitment (R/D)* causing atelectrauma can be seen as fully recruited alveoli filling the microscopic field (white circles, two of which are highlighted with black dotted line) **(a)** that collapse during expiration (red atelectatic areas highlighted by arrows) **(b)**. Histology shows open alveoli at inspiration with a shared alveolar wall highlighted by arrows **(c)** that collapse with expiration by alveolar wall folding, identified by arrows. Right – *Stress-focus (SF)* in areas with collapsed alveoli. SF adjacent to open alveolar ducts, causing alveolar duct *overdistension (OD)*. SF-induced OD causes excessive mechanical stress and strain on alveolar duct walls, resulting in damage to pulmonary parenchymal cells (Ghadiali and Gaver, 2008). Loss of surfactant function results in alveolar instability (Figure 3, R/D). Histology at exhalation in a rat Tween-induced surfactant deactivation model with a tidal volume of 6 ml/kg and a PEEP of 5 cm H_2_O. Note the heterogeneous collapse of alveoli causing areas of stress-focus (SF). Adjacent to these areas of SF are overdistended (OD) alveolar ducts (Kollisch-Singule et al., 2014b). Summary – R/D causes excessive normal or “peeling” stress as the adhered alveolar walls peel apart during inflation; both R/D and SF cause OD in adjacent open stable tissue. This excessive stress and strain on alveolar walls results in lung parenchymal cell death and is a major VILI mechanism. *Permissions obtained to reuse panels* **(a–d)** (Pavone et al., 2007). *Springer Nature license number 4766000130978.*

Mechanical ventilation of the acutely injured lung with altered alveolar opening and collapse time constants can cause tissue stress-failure by the following mechanisms: (1) alveolar R/D-induced excessive stress on the epithelial cells as the alveolar walls in apposition peel apart (Bilek et al., 2003), (2) stress-focusing in areas of open alveoli adjacent to collapsed or edema fill alveoli (Gattinoni et al., 2012; Cressoni et al., 2014; Retamal et al., 2014), and (3) collapsed tissue stretching and overdistending the shared walls of patent alveoli, as alveolar walls are interconnected (Figure 4) (Nieman et al., 2017b). Theoretically, if it were possible to minimize or prevent all of the above VILI mechanisms, ARDS associated mortality would be significantly reduced.

**FIGURE 5:**
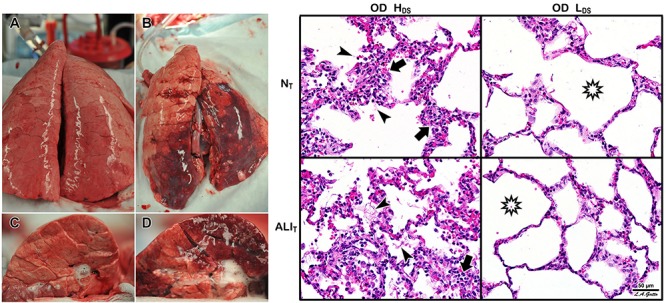
Heterogeneous Tween Injury in pigs ventilated for 6 h. A bronchoscope was used to deliver Tween to the dependent areas of the diaphragmatic or caudal lung lobes. Thus, the upper lobes would be normal homogeneously inflated tissue (i.e., would simulate the baby lung), and the dependent areas of the caudal lobe would model acutely injured tissue that would be either collapsed or unstable during tidal ventilation. Using this injury model we can determine whether a ventilation strategy protects either normal tissue (N_T_) or acutely injured lung tissue (ALI_T_), protects neither, or protects both. Two groups were studied. Both groups were subjected to a high static strain (Pplat = 40 cm H_2_O) hypothesized to be sufficient to cause overdistension (OD)-induced VILI to the baby lung (Acute Respiratory Distress Syndrome Network, 2000). One group was also subject to high dynamic strain (H_DS_) and the other to low dynamic strain (L_DS_). The dynamic strain was adjusted using the airway pressure release ventilation (APRV) mode by changing the expiratory duration, which changed tidal volume size. Left – Gross photographs of the whole lung **(A,B)** and cut surface **(C,D)** of the diaphragmatic lung lobe. The L_DS_ group **(A,C)** showed minimal damage in both N_T_ and ALI_T_ lung tissue (i.e., no dark red hepatized atelectasis). This was in contrast to the H_DS_ group, in which there was severe injury in both the N_T_ and ALI_T_ lung tissues **(B,D)**. Right – In the OD + H_DS_ group (OD + H_DS_), widespread histopathology typical of ARDS was seen, with inflammatory cell inflation (arrows) and fibrin deposits (arrowheads) in both the N_T_ and ALI_T_ lung tissue. In the OD + L_DS_ group, minimal histopathology was seen, and alveoli remained open (stars) in both N_T_ and ALI_T_ lung tissue. These data support Protti et al.’s (2013b) work and demonstrate that normal lung tissue is highly resistant to static strain-induced volutrauma. In addition, this study showed that acutely injured lung tissue is also resistant to volutrauma as long as dynamic strain remains low. Both normal and acutely injured lung tissue are highly susceptible to high alveolar R/D-induced VILI when under high inflation pressure (Jain et al., 2017). These data support the rapid progression of lung injury in a power-law fashion when high static and dynamic strain are combined (Hamlington et al., 2018).

In normal pigs ventilated for 54 h, Protti et al. (2013b) examined the impact of high static strain and several levels of dynamic strain. They demonstrated that *high static strain*, using elevated PEEP and minimal Vt, caused little damage, but when PEEP was reduced, producing a *high dynamic strain*, it caused pulmonary edema and death. In subsequent work they showed that high static strain did not merely act as a dam to prevent edema formation by altering the Starling forces (i.e., increasing the pulmonary interstitial pressure) (Effros and Parker, 2009) but rather preserved the integrity of the alveolar-capillary membrane (Figure 3, Endothelial Leakage) (Protti et al., 2013a). These findings were supported by Jain et al. (2017) using a porcine heterogeneous lung injury model. They subjected two study groups to high static strain (Pplat = 40 cm H_2_O) that was believed to be more than sufficient to cause volutrauma-induced VILI (Acute Respiratory Distress Syndrome Network, 2000; Sahetya et al., 2017). Following heterogeneous lung injury, the animals in the second group were also subjected to high dynamic strain. High static strain did not injure normal open tissue (baby lung), nor did it exacerbate acutely injured tissue. However, combining high static strain with high dynamic strain caused significant damage to both of these tissues (Figure 5). Further support for the contention that volutrauma of normal lung tissue is not a primary VILI mechanism comes from the Bates group, who showed that 4 h of mechanical ventilation in mice with high static strain was not associated with lung injury, but when it was combined with high dynamic strain, it caused VILI-induced tissue damage (Seah et al., 2011).

**FIGURE 6:**
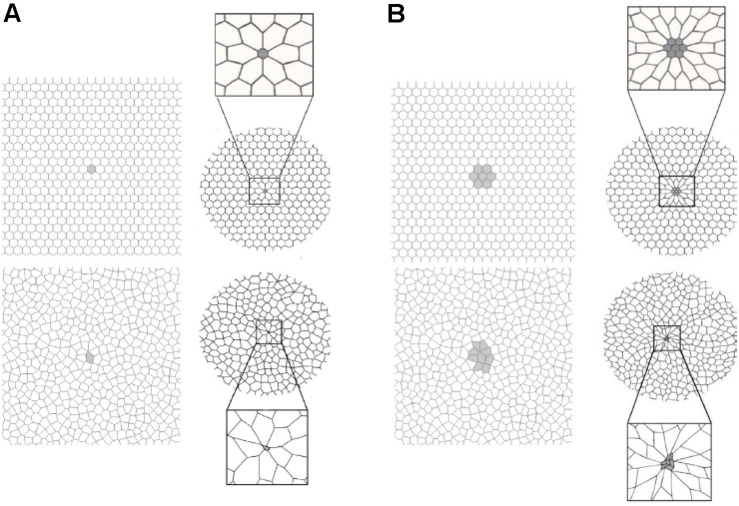
A two-dimensional finite element computational model of interconnected alveolar walls to study the impact of areas of stress-focus (S-F) on stress–strain relationships. Both a hexagonal honeycomb (top) and Voroni honeycombs (bottom) were studied with areas of S-F created by increasing the stiff regions in one **(A)** or nine **(B)** cells. These stiff regions simulate collapsed alveoli adjacent to open alveoli that create an S-F in acute respiratory distress syndrome (ARDS) patients (Figure 4) (Cressoni et al., 2014). The entire honeycomb structure was expanded and exposed to strains of 15, 30, 45, and 55% above the resting geometry. Some of the alveolar walls in the Voroni honeycomb were exposed to a S-F ∼16 times greater than that applied to uniformly expanded areas. This suggests that ventilation pressures considered safe in ARDS patients (<30 cm H_2_O) (Acute Respiratory Distress Syndrome Network, 2000) could be causing local stress concentration on some alveolar walls of approximately 48 cm H_2_O, with even higher stress in areas of S-F (Makiyama et al., 2014). *Permissions obtained from Elsevier. License 4699410294890.*

Protti et al. (2014) showed that a large dynamic strain (atelectrauma) is much more harmful to the normal lung than a large static strain (volutrauma), and when combined, the two work additively or synergistically to greatly accelerate tissue damage (Seah et al., 2011; Hamlington et al., 2016; Ruhl et al., 2019). In addition, the heterogeneous injury caused by ARDS establishes many areas of *stress-focus* between the open tissue and collapsed or unstable tissue and have been shown to double the stress and strain calculated for the entire lung (Cressoni et al., 2014). The impact that areas of stress-focus have on the forces generated on alveolar walls during ventilation was first described by Mead et al. (1970) and was more recently analyzed by Makiyama et al. (2014) using computer-simulated alveolar walls. This latter group demonstrated that a Pplat at the upper end of the “safe” level (30 cm H_2_O) could result in a local stress-focusing in individual alveolar walls of up to 48 cm H_2_O and could concentrate stress in an individual alveolar wall as much as 16-fold (Figure 6).

In summary, ARDS results in a heterogeneous loss of surfactant function (Figure 3, Surfactant Deactivation) and airway flooding (Figure 3, Alveolar Edema) that cause the lung to become *time and pressure dependent*, which means that the lung will collapse in a relatively short time at atmospheric pressure and will require a longer period of time to open even at high inflation pressures (Takahashi et al., 2015). Mechanical ventilation can exacerbate the initial ARDS-induced inflammatory injury (Figure 3, Endothelial Leakage, Surfactant Deactivation, and Alveolar Edema) by generating excessive stress and strain on alveolar and alveolar duct walls resulting from a collapsing and reopening of alveoli and heterogeneous areas of stress-focusing in open tissue adjacent to collapsed or edema-filled tissue (Figure 4).

**FIGURE 7:**
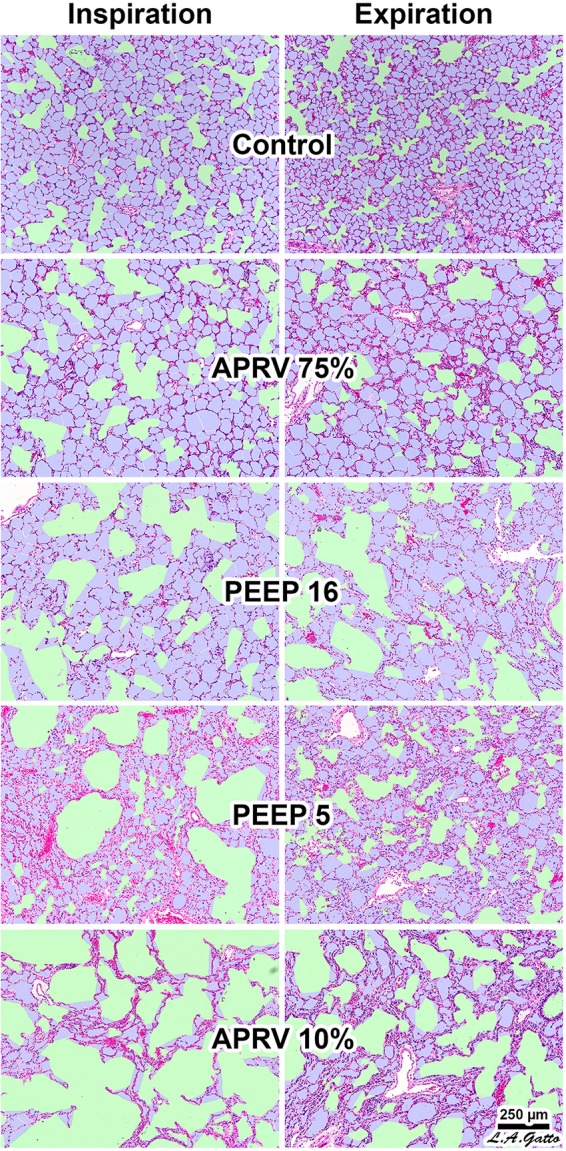
Rat lungs fixed at inspiration and expiration in normal lungs (**Control**) and in a Tween lavage ARDS model with four ventilation strategies: (1) the TCAV method setting airway pressure release ventilation (APRV) with the expiratory flow termination (E_FT_) at 75% of expiratory flow peak (E_FP_) (E_FT_/E_FP_ = 75%) (**APRV 75%**) (Habashi, 2005; Jain et al., 2016); (2) E_FT_/E_FP_ = 10% (**APRV 10%**), which significantly increased expiratory time; (3) controlled mandatory ventilation (CMV) with low tidal volume (6 mL/kg) with 5 cm H_2_O positive end-expiratory pressure (**PEEP 5**); or (4) CMV with low tidal volume (6 mL/kg) with 16 cm H_2_O PEEP (**PEEP 16**). **APRV 10%** (not the TCAV method) significantly increased expiratory duration, allowing sufficient time for alveoli to collapse. The conducting airways are depicted in green; alveoli in lilac; and remaining interstitium, blood vessels, and lymphatics in magenta. The size of each microanatomical area was quantified using computer image analysis. In the **Control** group all alveoli were inflated surrounding normally distended alveolar ducts. Following Tween-induced ARDS, alveolar ducts in all ventilation groups were increased in size. In the CMV groups alveolar ducts were overdistended at both inspiration and expiration in **PEEP16.** In the **PEEP5** group alveolar ducts were relatively normal size at expiration but greatly overdistended at inspiration with areas of alveolar collapse (i.e., high dynamic strain) at both inspiration and expiration. **APRV 10%** resulted in highly overdistended alveolar ducts at both inspiration and expiration with large areas of collapsed alveoli. **APRV 75%** (i.e., the TCAV method) resulted in the smallest increase in alveolar duct size, with uniformly open, homogeneously ventilated alveoli that were closest to those seen in the **Control** group (Kollisch-Singule et al., 2014b). *Permissions obtained from Elsevier. License 4732510649425.*

**FIGURE 8:**
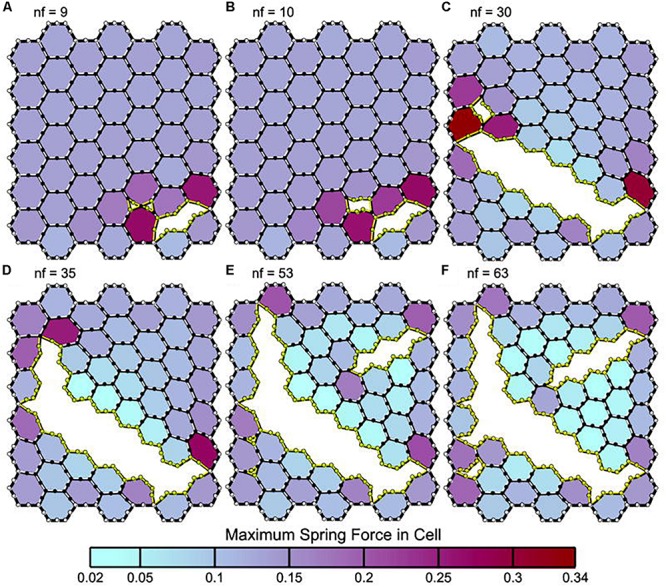
Panels **(A–F)** depict leak progression caused by an applied stretch force. A single node fails (nf) at each time point with nf indicating the number of nodes that have failed in each panel. Computational model of an epithelial monolayer to simulate leak progression due to overdistension. Leak progression in a 45-cell (hexagon) network caused by applied stretch (i.e., Vt). After the force required to initiate the leak was reached, the leak area increased at a constant rate as the force increased further (Hamlington et al., 2016). Atelectrauma caused the initial tears, after which volutrauma expanded those tears. The tears progressed in a rich-get-richer mechanism in which the likelihood of a tear getting larger increased with the size of the initial tear. This mechanism explains why atelectrauma appears to be essential to the initiation of VILI in a normal lung, and why atelectrauma and volutrauma act synergistically once VILI is underway (Hamlington et al., 2018). *Permissions obtained from Springer Nature. License 4699410825602.*

It is important to note that parenchymal overdistension following acute lung injury is a regional phenomenon and occurs only in open alveoli and alveolar ducts that are adjacent to the unstable or collapsed tissue (Figure 7, PEEP 16, PEEP 5, APRV 10%); it does not occur in homogeneously inflated acutely injured lung tissue (Figure 7, APRV 75%) (Nieman et al., 2017b). It has been shown that combining high pressure with alveolar instability greatly exacerbates tissue tearing in a rich-get-richer fashion (i.e., the larger the initial tear in the epithelial membrane the more that this tear will be expanded by increased airway pressure) (Figure 8) (Hamlington et al., 2016; Ruhl et al., 2019).

## Ventilating Within the Constraints of an Acutely Injured Lung

The ARDSnet method is a logical physiologically based lung ventilation strategy within the constraints of protecting a heterogeneously injured lung. The better strategy would be to eliminate these constraints by opening and stabilizing the acutely injured lung or, better yet, by applying a protective ventilation strategy early and never letting the lung collapse (Satalin et al., 2018). Gattinoni and Pesenti stressed that in a patient with ARDS the Vt should not be set by body weight (Vt/kg ratio) but rather by the size of the remaining normal open tissue at FRC: the baby lung (Vt/baby lung volume ratio) (Gattinoni and Pesenti, 2005). They hypothesized that the baby lung was the small amount of open tissue at end-expiration (i.e., FRC) surrounded by a large volume of collapsed tissue with very low compliance. Since the specific elastance [E_spec_ = transpulmonary pressure (Ptp)/Vt x baby lung volume, which is the airway pressure at which expiratory lung volume or FRC or baby lung volume doubles in size (i.e., when Vt/baby lung volume = 1)] if the baby lung is postulated to be normal, there would be a greater potential for overdistension in normal lung tissue because of the high compliance at any given level of static strain (Gattinoni and Pesenti, 2005). Both the potential of excessive stress (Ptp) to cause volutrauma and of excessive strain (Vt/end-expiratory lung volume) to cause atelectrauma are linked with the following equation:

Ptp(stress)=E×spec(Vt/babylungvolume).

(1) (Eq.1)(Gattinoni and Pesenti, 2005)

Gattinoni and Pesenti further concluded that the above equation suggests the baby lung must be treated gently, using low Vt, low Ptp, and proning so as not to cause volutrauma. Gently ventilating the lung should be protective, within the constraints of a heterogeneously injured lung (i.e., high E_spec_ and low baby lung volume).

The current strategy of lung protection is designed to minimize normal tissue overdistention (low Vt) and to stabilize (PEEP) injured tissue. Although the current hypothesis is that the primary mechanism of VILI is overdistension of the baby lung (Acute Respiratory Distress Syndrome Network, 2000), recent studies have demonstrated that high Ptp does not result in injury to normal lung tissue (Seah et al., 2011; Protti et al., 2013a, b, 2014; Jain et al., 2017). Since much of the lung remains collapsed and unstable using the ARDSnet method, there are numerous areas of stress-focus, as PEEP adjusted by changes in oxygenation may not be adequate to prevent R/D (Baumgardner et al., 2002; Boehme et al., 2015). Even with low Vt, tissue injury may occur regionally, since overdistension is not global but occurs in the microenvironment. Specifically, alveolar and alveolar duct overdistension occurs in open tissue surrounding a collapsed area of stress-focus or adjacent to areas of alveolar instability (Figures 4, 6, 7) (Mead et al., 1970; Cereda et al., 2011, 2013, 2016a; Kollisch-Singule et al., 2014b, 2018; Makiyama et al., 2014; Nieman et al., 2017b; Ruhl et al., 2019). Indeed, it has been shown that atelectasis, not high Vt, causes overdistension

in the adjacent patent alveoli and that alveolar size actually decreases with increased airway pressure (i.e., PEEP) if lung tissue is recruited by a mechanism of gas redistribution (Cereda et al., 2011, 2013, 2016a). It has also been shown that gas redistribution in the microenvironment is not only *pressure* dependent but also *time* dependent (i.e., the longer the pressure is applied, the better the gas redistribution) (Figure 7) (Kollisch-Singule et al., 2014b).

Animal studies have shown that the OLA can protect the lung from VILI even if ΔP is not reduced (Tojo et al., 2018). Recruiting the lung with PEEP has been shown to reduce tissue damage secondary to spontaneous breathing (SB) by two mechanisms: (1) the intensity of the SB is reduced via neuromechanical uncoupling, and (2) the reduced amount of atelectatic tissue decreases the volume of stress-focus areas (Morais et al., 2018). Other studies using animal models have also shown the physiologic and pathologic benefit of opening the acutely injured lung (Faridy et al., 1966; Webb and Tierney, 1974; Dreyfuss et al., 1988; Muscedere et al., 1994; Nakazawa et al., 2007; Nieman et al., 2015; Magalhaes et al., 2018). Additionally, animal studies have shown that to successfully implement the OLA, the RM and subsequent PEEP level must be applied properly, or the approach may actually increase lung damage. Farias et al. (2005) showed that an RM increased lung pathology if PEEP was not set sufficiently high to prevent recollapse of the newly opened tissue, a finding that is supported by direct *in vivo* observation of subpleural alveoli (Halter et al., 2003). In light of this evidence, discarding the OLA does not seem logical, since it is unclear whether the RCTs testing the OLA actually opened and stabilized the lung. A better strategy might be to identify ventilation strategies that are most likely to accomplish the goals of the OLA (Sahetya and Brower, 2017).

## A Physiologically Informed Strategy to Effectively Open and Stabilize the Lung

Surfactant deactivation and edema flooding with acute lung injury cause alveolar collapse; the result is very “sticky” and does not reopen easily (Figure 3) (Crotti et al., 2001; Gattinoni et al., 2003). This tissue has a very long opening time constant, and thus it will take an extended period of time at any given pressure to recruit these surfactant deficient and edematous tissues. Once the collapsed alveoli are recruited, the opposite problem develops; the newly opened alveoli have a very brief collapse time constant (Neumann et al., 1998a, b, 2000). Thus, alveoli collapse quickly (≤0.5 s) once a critical collapse airway pressure is reached (i.e., collapse is *time* and *pressure* dependent) (Kollisch-Singule et al., 2014a, b).

**FIGURE 9:**
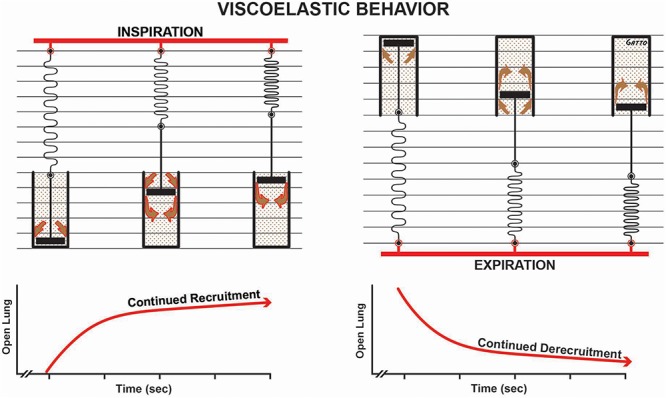
Viscoelastic behavior of alveolar recruitment and derecruitment described using a spring and dashpot model. During inspiration the spring is rapidly stretched, whereas the dash in the pot moves more slowly as fluid squeezes around it (brown arrows), in response to the applied force (i.e., tidal volume – red bar). The effect of this viscoelastic behavior is continual alveolar recruitment as long as inspiration is held (open lung/time curve). Viscoelastic behavior during expiration shows a rapid collapse of the spring and a much slower collapse of the dash in the pot. This is shown in the open lung/time curve as continuous alveolar collapse or derecruitment over time. Viscoelastic alveolar opening and collapse suggests that the longer inspiration is held, the more alveoli are recruited, and the shorter the expiratory time, the fewer the alveoli that collapse.

To effectively implement the OLA, the entire mechanical breath pattern (MB_P_), including all airway volumes, flows, pressure, rates, and the times during which they apply during inspiration and expiration, must be analyzed (Kollisch-Singule et al., 2014a). The current OLA ventilation strategies do not consider the pathophysiological changes that occur in alveolar opening and collapse time constants. Attempting to recruit large volumes of collapsed lung with a single RM over a very brief period of time is often not physiologically possible depending on the degree of surfactant damage and edema. A more effective strategy may be a ventilation method that “nudges” the lung open over an extended period of time (6–24 h) using a high mean airway pressure held for most of each breath. Likewise, the very fast alveolar collapse time constants are not considered when setting PEEP with expiratory durations in the 2- to 3-s range. A more effective strategy may be to use a ventilator method with a very brief (≤0.5 s) expiratory time while maintaining a level of time-controlled PEEP (TC-PEEP). Greatly limiting the expiratory duration (≤0.5 s) keeps the lung from emptying completely, maintaining a TC-PEEP that is related to expiratory duration and the collapse time constant of the lung. Thus, for any given collapse time constant, the shorter the expiratory time, the higher the TC-PEEP and vice versa. In addition, since alveolar collapse is viscoelastic in nature (Figure 9), the very short expiratory duration would work synergistically with TC-PEEP to prevent alveolar collapse – alveoli simply would not have sufficient time to derecruit (Nieman et al., 2019). Analysis of the change in alveolar opening and collapse time constants with acute lung injury suggests that the time component of the MB_P_ (Kollisch-Singule et al., 2014a) can be used to improve the ability to recruit and stabilize acutely injured lung tissue, which is necessary to successfully implement the OLA.

In addition to the time component of MB_P_, the dynamic physiology of alveolar R/D must also be understood to design a mechanical breath for the OLA. Alveoli and alveolar ducts inflate and deflate as a viscoelastic system (Denny and Schroter, 2000; Escolar and Escolar, 2004; Farias et al., 2005; Carvalho and Zin, 2011; Suki and Bates, 2011; Nieman et al., 2017b). The model most used to analyze viscoelastic behavior is the spring and dashpot (Figure 9). The most important thing to know about a viscoelastic system, in relation to lung opening and collapse, is that there is a time lag from when the force (inspiratory pressure) is applied until alveoli begins to open and a lag between when force is removed (expiratory pressure) and when alveoli begin to collapse. Thus, the longer the inspiratory time, the more lung tissue that will be recruited, and the shorter the expiratory time, the less lung tissue that will be allowed to recollapse (Figure 9). A collapsed airway inflates after the opening threshold pressure is reached, and the pressure then propagates down the airway, inflating more airways and alveoli. This process progresses in an avalanche manner with power-law distributions of both the size of and intervals between avalanches (Suki et al., 1994; Alencar et al., 2002). It has been postulated that as the lung opens, the increase in parenchymal tethering of airways (Broche et al., 2017) and alveolar interdependence improves lung function as a power-law function (Nieman et al., 2019). These new perspectives inform the quest for novel protective ventilation strategies. Indeed, our work in translational animal models and a meta-analysis of data on surgical intensive care unit (SICU) patients has shown that our time-controlled adaptive ventilation (TCAV) method, using airway pressure release ventilation (APRV) mode, is highly effective at keeping the lung open and stable, significantly reducing morbidity in translational animal models and reducing the ARDS incidence and mortality rates of SICU patients at high risk of developing ARDS (Roy et al., 2012; Andrews et al., 2013; Emr et al., 2013; Roy S.K. et al., 2013; Kollisch-Singule et al., 2014a, b, 2015a,b, 2017; Nieman et al., 2015, 2017a,b, 2018; Smith et al., 2015; Jain et al., 2016, 2017; Satalin et al., 2018; Silva et al., 2018; Mahajan et al., 2019).

Others have shown the importance of ventilation time on lung mechanics. Saddy et al. (2013) calculated a pressure-time product (PTP)/breath as the integral of the change in esophageal pressure over time. They found that, when using biphasic positive airway pressure, PTP significantly increased when the rate of time-cycled control breaths was at 50 breaths/min as compared to when it was 100 or 75 breaths/min. The energetics of ventilation also contain components of ventilator time. Power is defined as work per unit time and is thus equal to pressure x (volume/time) (Marini, 2018). Most of the energy applied to the lung during inflation is accounted for in elastic storage and airway resistance. It is postulated that the damage due to power is caused by the energy that is dissipated and unrecovered during exhalation. In addition, it is not just the power but also the changes in the microenvironment that result in tissue damage. Regional alveolar instability can develop during ARDS and can greatly amplify energy dissipation locally. Regional instability acts as a stress-focus, and once a microstrain threshold is reached, the unrecovered energy will cause a rapid progression of lung tissue injury in a power-law fashion (Hamlington et al., 2018).

## Eliminating Constraints of Ventilating the Acutely Injured Lung

It was recently shown in ARDS patients that low Vt and Pplat do not correlate well with reduced patient mortality, whereas low ΔP correlated strongly with improved survival (Amato et al., 2015). The critical lesson is that it is not the settings dialed into the ventilator (i.e., Vt, Pplat, and so on) that are key to improved survival, but rather the impact of these settings on lung physiology measured as a change in ΔP (Kollisch-Singule et al., 2014a, b, 2015b).

Using the ARDSnet method, the Vt was set at 6 cc/kg, the Pplat was set at < 30 cm H_2_O, and PEEP was set on a sliding oxygenation scale; the mechanism of improved mortality was assumed to be due to these parameters. The Amato study clearly demonstrated that the mechanism of increased survival was not these desired setting values, because none correlated with outcome (Amato et al., 2015). Lower ΔP, on the other hand, strongly correlated with reduced mortality. Respiratory system compliance (C_RS_) was used to calculate driving pressure (ΔP = Vt/C_RS_), which was shown to decrease in patients who survived, reflecting a desirable change in lung physiology caused by recruitment.

The ARDSnet method assumes that the constraints of ventilating a heterogeneously collapsed lung are unavoidable, in which case the Ptp is high due to the high E_spec_ value and low baby lung (FRC) volume. However, if the entire lung could be fully recruited, these constraints would be eliminated, E_spec_ reduced, and FRC increased, resulting in a significant reduction in the applied stress (i.e., Ptp) for any given Vt. Rahaman used a stress equation similar to Eq. 1 to analyze the mathematics of VILI (Rahaman, 2017). He concluded that stress increases for any given Vt and PEEP with an increase in respiratory rate (RR). This conclusion assumes that the clinician is constrained in ventilating a heterogeneously collapsed lung such that neither FRC (i.e., the size of the baby lung) nor E_spec_ can be changed. When these conditions are true, the conclusion that Vt, PEEP, and RR must be kept low to reduce stress is correct. Conversely, our perspective is the better strategy is to treat the lung by reinflating the collapsed tissue, increase FRC (eliminating the baby lung), and decreasing E_spec_. This combination will reduce lung stress (Ptp) during ventilation even at higher Vt, PEEP, and RR (Eq. 1).

Of course the strategy of “casting” the broken lung open until it heals would be clinically effective only with a ventilation strategy that could perform such a feat (Nieman et al., 2018). Unfortunately, the current OLA strategies have not been shown effective at opening and stabilizing the lung (Bhattacharjee et al., 2018; Cui et al., 2019; Hodgson et al., 2019; Kang et al., 2019; Zheng et al., 2019), and the recent ART trial showed an increase in mortality using the OLA (Brower et al., 2004; Meade et al., 2008; Mercat et al., 2008; Cavalcanti et al., 2017).

## The TCAV Method to Open and Stabilize the Acutely Injured Lung

Our TCAV method using the APRV mode has been discussed in detail elsewhere (Habashi, 2005; Jain et al., 2016; Nieman et al., 2018, 2019; Kollisch-Singule et al., 2019). Briefly, TCAV consists of an extended (4–5 s) open valve continuous positive airway pressure (CPAP) phase with a very short (≤0.5 s) release phase. The inspiratory:expiratory ratio is ∼10:1. The open valve allows the patient to spontaneously breath (inspiration or expiration) with little resistance. Tidal volume (Vt) is not set but rather is a product of the CPAP level and lung compliance. A heterogeneously collapsed lung will have a very low Vt because the compliance will be low, but as the lung gradually recruits over time, the compliance will increase and so will the Vt. Since the Vt is set based on lung compliance, a high Vt will never be delivered to a non-compliant collapsed lung using the TCAV method. Thus, the Vt size is personalized and adaptive as the patient’s lung gets better or worse, directed by changes in lung compliance. The extended CPAP time will gradually nudge viscoelastic alveoli open over several hours until the lung is fully inflated. The newly recruited alveoli will be prevented from recollapse through the use of a very short expiratory duration (release phase). Expiratory time is very short (≤0.5 s), and thus the lung is reinflated (CPAP phase) before it has time to completely empty, maintaining a TC-PEEP. The very short expiratory time is not sufficient for viscoelastic alveoli to collapse and when combined with the TC-PEEP is highly effective at preventing alveolar derecruitment.

Our group has investigated the physiological impact of the TCAV method in both mechanistic and efficacy animal studies (Roy et al., 2012; Emr et al., 2013; Roy S.K. et al., 2013; Roy S. et al., 2013; Kollisch-Singule et al., 2014a, b, 2015a,b, 2017; Smith et al., 2015; Silva et al., 2018) and in a meta-analysis of data on SICU patients (Andrews et al., 2013). In addition, the TCAV method is the primary ventilator strategy used at the R Adam Cowley Shock Trauma Center in Baltimore, with well over 1,000,000 h of ventilator time. Below, we discuss the results from our animal data and our clinical statistical analysis as evidence for the TCAV method’s mechanisms and efficacy.

**FIGURE 10:**
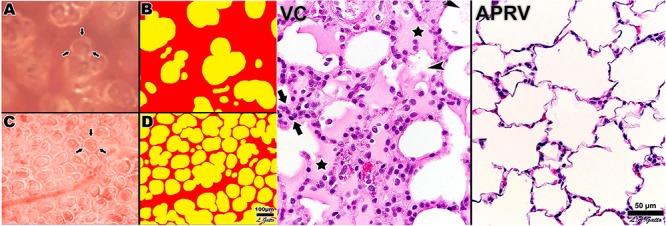
Left – Subpleural alveoli seen using *in vivo* microscopy in a rat hemorrhagic shock-induced ARDS model ventilated with volume cycled ventilation (*VC*; **A,B**) or airway pressure release ventilation (*APRV*) using the time controlled adaptive ventilation (TCAV) method (*APRV*; **C,D**). Individual alveoli are shown by arrows. Inflated alveoli were color coded yellow, and alveolar number, size, and surface area were measured by computer image analysis in each group. TCAV significantly improved alveolar patency and stability as compared with the VC group. Right – The *APRV* group delivered using the TCAV method stabilized alveoli that was associated with a significant reduction in lung histopathology as evidenced by open alveoli and reduced intra-alveolar edema (purple areas) as compared with the collapsed and edema-filled alveoli (stars), fibrinous deposits in the air compartment (arrowheads), and white cell infiltration (between arrows) in the *VC* group (Roy S.K. et al., 2013). *Permissions obtained from Wolters Kluwer Health, Inc. License 4699411295724.*

**FIGURE 11:**
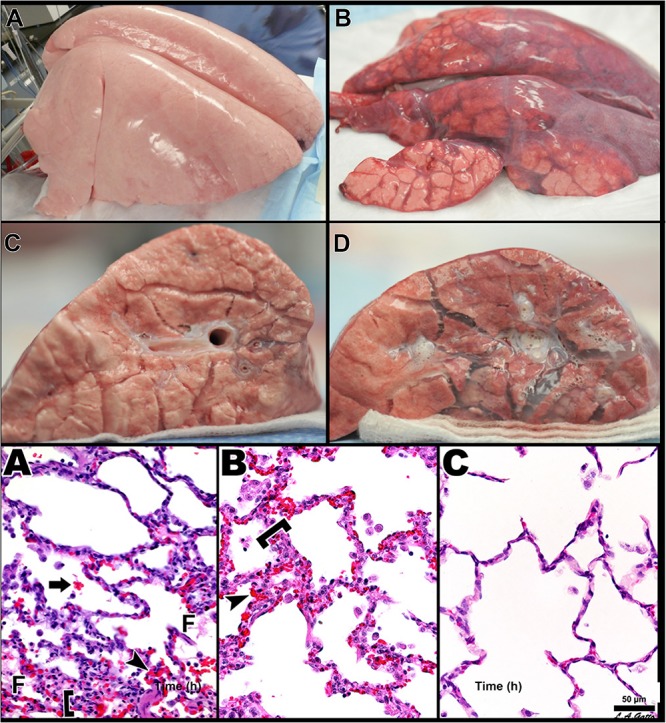
Three groups of pigs were ventilated for 48 h with a clinically applicable, high-fidelity porcine peritoneal sepsis (PS) and gut ischemia/reperfusion (I/R) acute respiratory distress syndrome (ARDS) model: (1) *Control* group without PS + I/R injury ventilated with a tidal volume (Vt) of 10 cc/kg and a positive end-expiratory pressure (PEEP) of 5 cm H_2_O. (2) *ARDSnet* group using a low-Vt ventilation method, applied following PS + I/R injury at the time point at which the animal reached the oxygen saturation limit listed in the ARDSnet protocol, with PEEP and FiO_2_ adjusted by oxygenation (Acute Respiratory Distress Syndrome Network, 2000). (3) Time-controlled adaptive ventilation (*TCAV*) method using the airway pressure release ventilation (APRV) mode applied immediately after PS + I/R injury (Habashi, 2005; Jain et al., 2016). **Upper** – Gross lung photographs after 48 h of ventilation following PS + I/R injury at necropsy (*Control* not shown). In the *TCAV* group **(A,B)**, lungs inflated fully to near total lung capacity at 25 cm H_2_O without any gross atelectasis. The cut surface of the diaphragmatic lobe also showed no interstitial or airway edema and no atelectasis. Lungs in the *ARDSnet* group **(C,D)** also inflated to 25 cm H_2_O showed low lung volume with heterogeneous collapse and atelectasis and were wet and “boggy.” The cut surface showed both interstitial and airway edema. **Lower** – Photomicrographs of representative lung sections of specimens from the *Control* **(A)**, *ARDSnet* **(B)**, and *TCAV* **(C)** groups each at 40× magnification. F, fibrinous deposit in the air compartment; arrow, blood in alveolus; arrowhead, congested alveolar capillary; bracket, thickened alveolar wall. **(A)** *Control*: animals received 48 h of mechanical ventilation without PS + I/R injury. Specimen shows typical early acute lung injury pathology including fibrinous deposits, blood in alveolus, congested capillaries, and thickened alveolar walls. **(B)** *ARDSnet*: animals received PS + I/R injury, and LVt ventilation was applied after the onset of acute lung injury (i.e., PaO_2_/FiO_2_ ratio <300). Specimen shows typical advanced acute lung injury pathology including fibrinous deposits, blood in alveolus, congested capillaries, leukocyte infiltration, and thickened alveolar walls. **(C)** *TCAV*: specimen shows normal pulmonary architecture, alveoli are well-expanded and thin walled, and there are no exudates (Roy S. et al., 2013). *Permissions obtained from Wolters Kluwer Health, Inc. License 4699411460832.*

**FIGURE 12:**
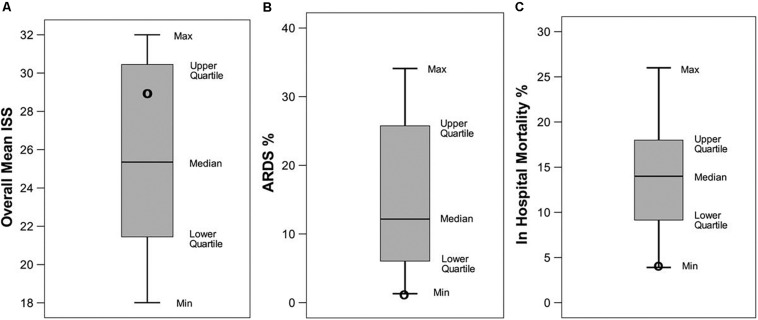
A systematic review of data from trauma patients who were ventilated with either conventional mechanical ventilation (CMV) or the time-controlled adaptive ventilation (TCAV) method using the airway pressure release ventilation (APRV) mode. Boxplots are mean and standard error for **(A)** Injury Severity Score (ISS), **(B)** percentage of patients who developed ARDS (ARDS%), and **(C)** in-hospital mortality (%) from 16 published papers using standard of care CMV. Black circles indicate the results in each category using the TCAV method at the *R Adam Cowley Shock Trauma Center* in Maryland. Although patients in the TCAV group were in the high-ISS range, they had the lowest levels of both ARDS incidence (ARDS%) and in-hospital mortality (%) as compared with patients ventilated using CMV (Andrews et al., 2013). *Permissions obtained from Wolters Kluwer Health, Inc. License 4699420119915.*

In rat VILI and hemorrhagic shock models, it was shown that ventilation for 6 h using the TCAV method was superior to volume-controlled ventilation (VCV; Vt 10 ml/kg, PEEP 0.5 cm H_2_O) at preventing the development of ARDS and that lung protection was associated with stabilization of alveoli (Figure 10) (Emr et al., 2013; Roy S.K. et al., 2013). The TCAV method was also shown to be lung protective in a preterm piglet model of infant respiratory distress syndrome (Kollisch-Singule et al., 2017). These data were supported by mechanistic studies showing the ability of the TCAV method to normalize alveolar and alveolar duct microanatomy (Kollisch-Singule et al., 2014b) (Figure 7) and to reduce dynamic alveolar strain (Kollisch-Singule et al., 2014a, 2015b; Smith et al., 2015). We developed a 48-hr clinically applicable, high-fidelity, porcine peritoneal sepsis (PS) plus gut ischemia/reperfusion (I/R), multiple organ dysfunction syndrome (MODS) and ARDS model. In three studies using this clinically applicable model, we demonstrated that the TCAV method was superior to VC or the ARDSnet method at blocking progressive acute lung injury and preventing ARDS development (Figure 11) (Roy et al., 2012; Roy S. et al., 2013; Kollisch-Singule et al., 2015a). In addition, we have shown that surfactant proteins A and B are both better preserved with the TCAV as compared with the ARDSnet method, suggesting that there is sufficient lung volume change with TCAV to preserve stretch-induced surfactant release (Roy et al., 2012; Emr et al., 2013; Roy S.K. et al., 2013; Roy S. et al., 2013; Kollisch-Singule et al., 2015a). Lastly, in a statistical analysis of data from SICU patients, the TCAV method was associated with a significant reduction in ARDS incidence and mortality as compared to standard of care ventilation in 15 SICUs (Figure 12) (Andrews et al., 2013).

It is important to remember that if the acutely injured lung can be fully recruited, it will remain *time* and *pressure* dependent (i.e., will collapse quickly at atmospheric pressure) for a period of hours to days (Takahashi et al., 2015). Once the acutely injured lung is opened, the ventilator pressure/time support necessary to keep it open cannot be reduced until the lung “heals.” Gradually pulmonary edema will be reabsorbed and surfactant function restored (Vazquez de Anda et al., 2000). As more and more lung tissue is recruited, reestablishing alveolar interdependence (Figure 6) (Mead et al., 1970; Makiyama et al., 2014) and parenchymal tethering (Perun and Gaver, 1995), the lung will once again become stable at atmospheric airway pressure, and the weaning process can begin (Mead et al., 1970; Perun and Gaver, 1995; Makiyama et al., 2014).

Recent RCTs have shown mixed results in ARDS patients using APRV (but not utilizing the TCAV method). Zhou et al. (2017) randomized patients with a PaO_2_/FiO_2_ (P/F) ratio < 200 to either ARDSnet LVt or APRV with TCAV-like settings. The APRV group demonstrated significant decreases in length of ICU stay, tracheostomy requirement, ventilator days, and mortality. The APRV group also had significant reductions in the need for prone positioning, sedation, and neuromuscular blockade.

Lalgudi Ganesan et al. (2018) conducted an RCT in children with ARDS, who were randomized to receive either standard LVt strategy or APRV. The APRV group was shown not to be lung protective, and the trial was terminated early. We postulate the failure was due to fundamental misconceptions as to the MB_P_ necessary to protect the lung. A key objective in our TCAV method is to gradually nudge the lung open. In the Ganesan study, the CPAP pressure was reduced to generate a low Vt, and thus the CPAP level was likely not above the lung critical opening pressure, meaning the lung was not inflated. In the TCAV method, Vt is not set (see TCAV description above), so an increase in release volume (analogous to Vt) during the release phase and an increase in C_RS_ are positive signs and indictive of lung recruitment (Roy et al., 2012; Roy S. et al., 2013; Kollisch-Singule et al., 2015a). If the ventilation strategy used does not actually open the lung, the VILI mechanisms associated with heterogeneous ventilation will not be prevented (Figure 4). We postulate that this is also the reason for the failed RCTs using an RM and titrated PEEP (Brower et al., 2004; Meade et al., 2008; Mercat et al., 2008; Cavalcanti et al., 2017).

Hirshberg et al. (2018) conducted an RCT in adults, and similarly to the Ganesan study, targeted a Vt at ∼6 mL/kg. The study was stopped early, in part because the Vt often exceeded 12 mL/kg, even though there were no significant differences between groups in pneumothorax, sedation, vasoactive medications, P/F ratio, or outcome (Hirshberg et al., 2018). As stated above, when the TCAV method is used, an increase in Vt is indicative of lung recruitment and is a positive effect. Thus, one of the critical protective mechanisms of the TCAV method, the ability to fully open the lung, was not incorporated into the APRV setting in either the Hirshberg or Ganesan study. The conclusion from these clinical studies should not be that “APRV is not lung protective” but rather that the Zhou (Zhou et al., 2017) and TCAV methods (Andrews et al., 2013) are superior to the Ganesan (Lalgudi Ganesan et al., 2018) and Hirshberg (Hirshberg et al., 2018) methods for lung protection. Two recent statistical reviews and meta-analyses of RCTs have shown the APRV mode is associated with a mortality benefit, improved oxygenation, and a greater number of ventilator-free days when compared with conventional ventilation strategies, without any negative hemodynamic impact or higher risk of barotrauma (Carsetti et al., 2019; Lim and Litton, 2019).

**FIGURE 13:**
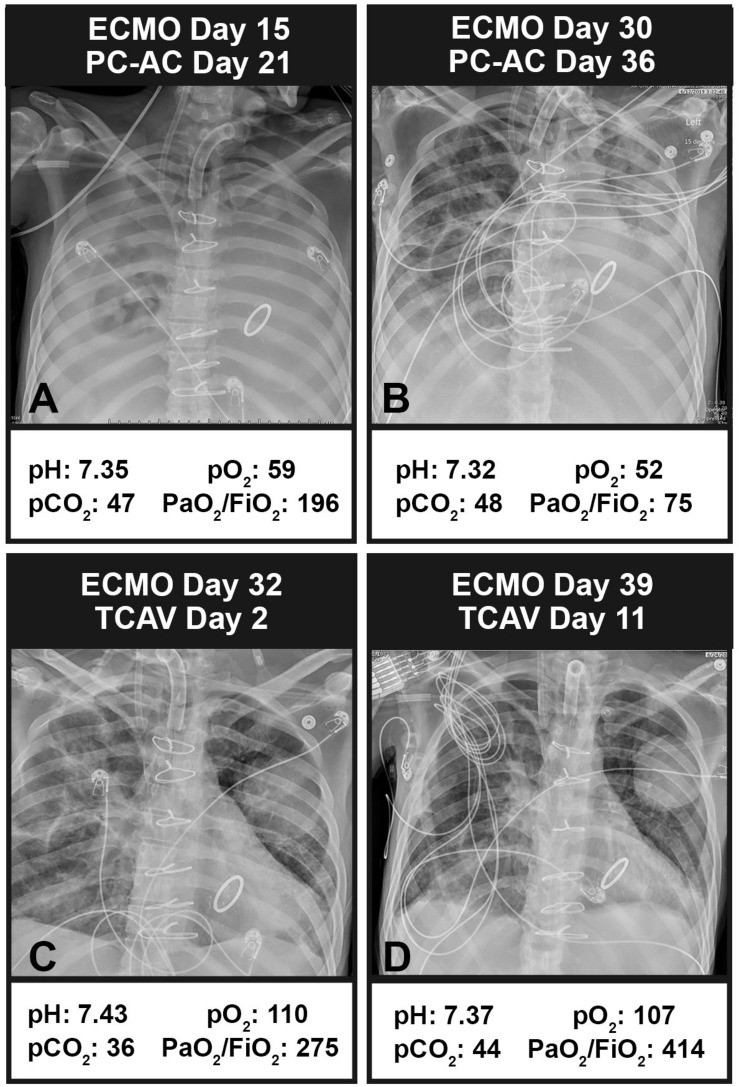
Chest X-rays (CXRs) and blood gases in a patient with respiratory failure on extracorporeal membrane oxygenation (ECMO). **(A)** CXR and blood gases (arterial pH, PCO_2_, and PO_2_ with the calculated PaO_2_/FiO_2_ ratio) at 15 days on ECMO and protective ARDSnet pressure-control assist-control ventilation (PC-AC). Bilateral infiltrates can be seen throughout the lung with a PaO_2_/FiO_2_ ratio of 196. **(B)** After 30 days on ECMO and PC-AC, the patient showed some clearing in the upper right quadrant but a worsening PaO_2_/FiO_2_ ratio of 75. **(C)** Two days after conversion to the TCAV method, the patient showed marked improvement in CXR and PaO_2_/FiO_2_ ratio (275). **(D)** After 11 days on the TCAV method, the patient showed a cleared CXR and normalized PaO_2_/FiO_2_ ratio (414). The patient came off of ECMO and was extubated. *Written informed consent obtained for the use of these images.*

An example of the TCAV method’s effectiveness when applied to a patient on extracorporeal membrane oxygenation (ECMO) with respiratory failure is seen in Figure 13. A 35-year-old man with a history of systemic lupus erythematous, with catastrophic antiphospholipid syndrome, underwent a coronary artery bypass graft with mitral valve repair and was placed on protective pressure-control assist-control ventilation (PC-AC) with protective settings using the ARDSnet method. Chest X-rays (CXRs) on PC-AC for 21 days, 15 of those days on ECMO, showed widespread infiltrates (Figure 13A). Another 15 days on ECMO (30 days on ECMO total) and PC-AC showed minimal change in CXRs and worsening lung function assessed by P/F ratio (A: 196, B: 75) (Figure 13B). Conversion to the TCAV method for 2 days resulted in a marked improvement in CXRs and P/F ratio (275) (Figure 13C). After 11 days on TCAV, the patient had a clear CXR and normal P/F ratio (414) (Figure 13D). Figure 13D was 6 h prior to decannulation and liberation from ECMO. After 39 days on PC-AC with worsening lung function, it took only 2 days on the TCAV method to reopen and stabilize the patient’s lung, and after 11 days the patient was disconnected from ECMO, extubated, and removed from mechanical ventilation.

## Conclusion

Current protective ventilation strategies have not significantly reduced ARDS-related mortality in the 20 years since the ARMA study, possibly because strategies are constrained to ventilating the heterogeneously collapsed and unstable lung. In addition, current protective ventilation strategies have not considered all of the physiological parameters necessary to protect the acutely injured lung. Alveoli and alveolar ducts open and collapse as a viscoelastic system such that there is a time lag between when the airway pressure is applied and alveoli open, and between when the airway pressure is removed and alveoli collapse. Loss of pulmonary surfactant function, a key ARDS pathology, amplifies these viscoelastic properties, rendering the lung *time* and *pressure* dependent, such that it takes an extended time at the higher pressure for lung tissue to open and a very brief time, even with an elevated airway pressure, for the lung to recollapse.

This knowledge suggests that to eliminate VILI the ventilation strategy must open and stabilize the lung. Current ventilation strategies attempting this OLA have not reduced mortality in clinical trials. Since alveolar volume change is viscoelastic in nature, the *time* at both inspiration and expiration would be critical to accomplishing the OLA goals. We have developed a time-controlled adaptive ventilation TCAV method using the APRV mode and have shown that it is highly effective at opening and stabilizing the lung, which significantly reduces VILI-induced lung damage in animal ARDS models. We have also shown that preemptive application of the TCAV method in trauma patients significantly reduces ARDS incidence and mortality. Recent RCTs using the APRV mode but not adjusted with the TCAV method have shown mixed results, suggesting that properly adjusted APRV is critical to clinical success.

## Author Contributions

GN drafted the manuscript. HA-K, MK-S, JS, SB, GT, PA, MM, LG, and NH critically revised the manuscript. All authors read and approved the final manuscript.

## Conflict of Interest

GN, HA-K, MK-S, JS, LG, PA, MM, and NH have lectured for Intensive Care On-line Network, Inc. (ICON). NH is the founder of ICON, of which PA and MM are employees. The authors maintain that industry had no role in the design and conduct of the study; the collection, management, analysis, or interpretation of the data; nor the preparation, review, or approval of the manuscript. The remaining authors declare that the research was conducted in the absence of any commercial or financial relationships that could be construed as a potential conflict of interest.

## References

[B1] Albert R. K. (2012). The role of ventilation-induced surfactant dysfunction and atelectasis in causing acute respiratory distress syndrome. *Am. J. Respir. Crit. Care Med.* 185 702–708. 22227381 10.1164/rccm.201109-1667PP

[B2] Alencar A. M. Arold S. P. Buldyrev S. V. Majumdar A. Stamenovic D. Stanley H. E. (2002). Dynamic instabilities in the inflating lung. *Nature* 417 809–811. 10.1038/417809b 12075340

[B3] Amato M. B. Meade M. O. Slutsky A. S. Brochard L. Costa E. L. Schoenfeld D. A. (2015). Driving pressure and survival in the acute respiratory distress syndrome. *N. Engl. J. Med.* 372 747–755. 10.1056/NEJMsa1410639 25693014

[B4] Andrews P. L. Sadowitz B. Kollisch-Singule M. Satalin J. Roy S. Snyder K. (2015). Alveolar instability (atelectrauma) is not identified by arterial oxygenation predisposing the development of an occult ventilator-induced lung injury. *Intensive Care Med. Exp.* 3:16. 10.1186/s40635-015-0054-1 26215818 PMC4480795

[B5] Andrews P. L. Shiber J. R. Jaruga-Killeen E. Roy S. Sadowitz B. O’Toole R. V. (2013). Early application of airway pressure release ventilation may reduce mortality in high-risk trauma patients: a systematic review of observational trauma ARDS literature. *J. Trauma Acute Care Surg.* 75 635–641. 24064877 10.1097/TA.0b013e31829d3504

[B6] Acute Respiratory Distress Syndrome Network (2000). Ventilation with lower tidal volumes as compared with traditional tidal volumes for acute lung injury and the acute respiratory distress syndrome. The acute respiratory distress syndrome network. *N. Engl. J. Med.* 342 1301–1308. 10.1056/nejm200005043421801 10793162

[B7] Ashbaugh D. G. Bigelow D. B. Petty T. L. Levine B. E. (1967). Acute respiratory distress in adults. *Lancet* 2 319–323. 4143721 10.1016/s0140-6736(67)90168-7

[B8] Baumgardner J. E. Markstaller K. Pfeiffer B. Doebrich M. Otto C. M. (2002). Effects of respiratory rate, plateau pressure, and positive end-expiratory pressure on PaO2 oscillations after saline lavage. *Am. J. Respir. Crit. Care Med.* 166(12 Pt 1) 1556–1562. 10.1164/rccm.200207-717oc 12406831

[B9] Bellani G. Laffey J. G. Pham T. Fan E. Brochard L. Esteban A. (2016). Epidemiology, patterns of care, and mortality for patients with acute respiratory distress syndrome in intensive care units in 50 countries. *J. Am. Med. Assoc.* 315 788–800. 10.1001/jama.2016.0291 26903337

[B10] Bergez M. Fritsch N. Tran-Van D. Saghi T. Bounkim T. Gentile A. (2019). PEEP titration in moderate to severe ARDS: plateau versus transpulmonary pressure. *Ann. Intensive Care.* 9:81. 10.1186/s13613-019-0554-3 31312921 PMC6635540

[B11] Bhattacharjee S. Soni K. D. Maitra S. (2018). Recruitment maneuver does not provide any mortality benefit over lung protective strategy ventilation in adult patients with acute respiratory distress syndrome: a meta-analysis and systematic review of the randomized controlled trials. *J. Intensive Care* 6:35. 10.1186/s40560-018-0305-9 PMC6019312 29983985

[B12] Bilek A. M. Dee K. C. Gaver D. P. (2003). Mechanisms of surface-tension-induced epithelial cell damage in a model of pulmonary airway reopening. *J. Appl. Physiol.* 94 770–783. 10.1152/japplphysiol.00764.2002 12433851

[B13] Blanch L. Villagra A. Sales B. Montanya J. Lucangelo U. Lujan M. (2015). Asynchronies during mechanical ventilation are associated with mortality. *Intensive Care Med.* 41 633–641. 10.1007/s00134-015-3692-6 25693449

[B14] Boehme S. Bentley A. H. Hartmann E. K. Chang S. Erdoes G. Prinzing A. (2015). Influence of inspiration to expiration ratio on cyclic recruitment and derecruitment of atelectasis in a saline lavage model of acute respiratory distress syndrome. *Crit. Care Med.* 43 e65–e74. 10.1097/CCM.0000000000000788 25513783

[B15] Borges J. B. Okamoto V. N. Matos G. F. Caramez M. P. Arantes P. R. Barros F. (2006). Reversibility of lung collapse and hypoxemia in early acute respiratory distress syndrome. *Am. J. Respir. Crit. Care Med.* 174 268–278. 10.1164/rccm.200506-976oc 16690982

[B16] Broche L. Perchiazzi G. Porra L. Tannoia A. Pellegrini M. Derosa S. (2017). Dynamic mechanical interactions between neighboring airspaces determine cyclic opening and closure in injured lung. *Crit. Care Med.* 45 687–694. 10.1097/CCM.0000000000002234 28107207 PMC5702254

[B17] Brower R. G. Lanken P. N. MacIntyre N. Matthay M. A. Morris A. Ancukiewicz M. (2004). Higher versus lower positive end-expiratory pressures in patients with the acute respiratory distress syndrome. *N. Engl. J. Med.* 351 327–336. 10.1056/nejmoa032193 15269312

[B18] Brower R. G. Shanholtz C. B. Fessler H. E. Shade D. M. White P. Jr. Wiener C. M. (1999). Prospective, randomized, controlled clinical trial comparing traditional versus reduced tidal volume ventilation in acute respiratory distress syndrome patients. *Crit. Care Med.* 27 1492–1498. 10.1097/00003246-199908000-00015 10470755

[B19] Brun-Buisson C. Minelli C. Bertolini G. Brazzi L. Pimentel J. Lewandowski K. (2004). Epidemiology and outcome of acute lung injury in European intensive care units. Results from the ALIVE study. *Intensive Care Med.* 30 51–61. 10.1007/s00134-003-2022-6 14569423

[B20] Burkhardt A. (1989). Alveolitis and collapse in the pathogenesis of pulmonary fibrosis. *Am. Rev. Respir. Dis.* 140 513–524. 10.1164/ajrccm/140.2.513 2669580

[B21] Burki N. K. Lee L. Y. (2010). Mechanisms of dyspnea. *Chest* 138 1196–1201. 10.1378/chest.10-0534 21051395 PMC2972628

[B22] Cabrera-Benitez N. E. Laffey J. G. Parotto M. Spieth P. M. Villar J. Zhang H. (2014). Mechanical ventilation-associated lung fibrosis in acute respiratory distress syndrome: a significant contributor to poor outcome. *Anesthesiology* 121 189–198. 10.1097/ALN.0000000000000264 24732023 PMC4991945

[B23] Carsetti A. Damiani E. Domizi R. Scorcella C. Pantanetti S. Falcetta S. (2019). Airway pressure release ventilation during acute hypoxemic respiratory failure: a systematic review and meta-analysis of randomized controlled trials. *Ann. Intensive Care* 9:44. 10.1186/s13613-019-0518-7 30949778 PMC6449410

[B24] Carvalho A. R. Zin W. A. (2011). Respiratory system dynamical mechanical properties: modeling in time and frequency domain. *Biophys. Rev.* 3:71. 10.1007/s12551-011-0048-5 28510005 PMC5418399

[B25] Caser E. B. Zandonade E. Pereira E. Gama A. M. Barbas C. S. (2014). Impact of distinct definitions of acute lung injury on its incidence and outcomes in Brazilian ICUs: prospective evaluation of 7,133 patients^∗^. *Crit. Care Med.* 42 574–582. 10.1097/01.ccm.0000435676.68435.56 24158166

[B26] Cavalcanti A. B. Suzumura E. A. Laranjeira L. N. Paisani D. M. Damiani L. P. Guimaraes H. P. (2017). Effect of lung recruitment and titrated positive end-expiratory pressure (PEEP) vs low PEEP on mortality in patients with acute respiratory distress syndrome: a randomized clinical trial. *JAMA* 318 1335–1345. 10.1001/jama.2017.14171 28973363 PMC5710484

[B27] Cereda M. Emami K. Kadlecek S. Xin Y. Mongkolwisetwara P. Profka H. (2011). Quantitative imaging of alveolar recruitment with hyperpolarized gas MRI during mechanical ventilation. *J. Appl. Physiol.* 110 499–511. 10.1152/japplphysiol.00841.2010 21127207 PMC3043787

[B28] Cereda M. Emami K. Xin Y. Kadlecek S. Kuzma N. N. Mongkolwisetwara P. (2013). Imaging the interaction of atelectasis and overdistension in surfactant-depleted lungs. *Crit. Care Med.* 41 527–535. 10.1097/CCM.0b013e31826ab1f2 23263577 PMC3557664

[B29] Cereda M. Xin Y. Hamedani H. Bellani G. Kadlecek S. Clapp J. (2017). Tidal changes on CT and progression of ARDS. *Thorax* 72 981–989. 10.1136/thoraxjnl-2016-209833 28634220 PMC5738538

[B30] Cereda M. Xin Y. Hamedani H. Clapp J. Kadlecek S. Meeder N. (2016a). Mild loss of lung aeration augments stretch in healthy lung regions. *J. Appl. Physiol.* 120 444–454. 10.1152/japplphysiol.00734.2015 26662053 PMC4754623

[B31] Cereda M. Xin Y. Meeder N. Zeng J. Jiang Y. Hamedani H. (2016b). Visualizing the propagation of acute lung injury. *Anesthesiology* 124 121–131. 10.1097/ALN.0000000000000916 26536308 PMC4681653

[B32] Chen Z. L. Chen Y. Z. Hu Z. Y. (2014). A micromechanical model for estimating alveolar wall strain in mechanically ventilated edematous lungs. *J. Appl. Physiol.* 117 586–592. 10.1152/japplphysiol.00072.2014 24947025

[B33] Coruh B. Luks A. M. (2014). Positive end-expiratory pressure. When more may not be better. *Ann. Am. Thorac. Soc.* 11 1327–1331. 10.1513/annalsats.201404-151cc 25343201

[B34] Cressoni M. Cadringher P. Chiurazzi C. Amini M. Gallazzi E. Marino A. (2014). Lung inhomogeneity in patients with acute respiratory distress syndrome. *Am. J. Respir. Crit. Care Med.* 189 149–158. 10.1164/rccm.201308-1567OC 24261322

[B35] Cressoni M. Chiumello D. Algieri I. Brioni M. Chiurazzi C. Colombo A. (2017). Opening pressures and atelectrauma in acute respiratory distress syndrome. *Intensive Care Med.* 43 603–611. 10.1007/s00134-017-4754-8 28283699

[B36] Crotti S. Mascheroni D. Caironi P. Pelosi P. Ronzoni G. Mondino M. (2001). Recruitment and derecruitment during acute respiratory failure: a clinical study. *Am. J. Respir. Crit. Care Med.* 164 131–140. 10.1164/ajrccm.164.1.2007011 11435251

[B37] Cui Y. Cao R. Wang Y. Li G. (2019). Lung recruitment maneuvers for ARDS patients: a systematic review and meta-analysis. *Respiration* 1–13. 10.1159/000501045 [Epub ahead of print]. 31330508

[B38] de Anda G. F. Gommers D. Verbrugge S. J. De Jaegere A. Lachmann B. (2000). Mechanical ventilation with high positive end-expiratory pressure and small driving pressure amplitude is as effective as high-frequency oscillatory ventilation to preserve the function of exogenous surfactant in lung-lavaged rats. *Crit. Care Med.* 28 2921–2925. 10.1097/00003246-200008000-00039 10966272

[B39] Del Sorbo L. Goligher E. C. McAuley D. F. Rubenfeld G. D. Brochard L. J. Gattinoni L. (2017). Mechanical ventilation in adults with acute respiratory distress syndrome. Summary of the experimental evidence for the clinical practice guideline. *Ann. Am. Thorac. Soc.* 14(Suppl. 4) S261–S270. 28985479 10.1513/AnnalsATS.201704-345OT

[B40] Denny E. Schroter R. C. (2000). Viscoelastic behavior of a lung alveolar duct model. *J. Biomech. Eng.* 122 143–151. 10.1115/1.429644 10834154

[B41] Dreyfuss D. Soler P. Basset G. Saumon G. (1988). High inflation pressure pulmonary edema. Respective effects of high airway pressure, high tidal volume, and positive end-expiratory pressure. *Am. Rev. Respir. Dis.* 137 1159–1164. 10.1164/ajrccm/137.5.1159 3057957

[B42] Effros R. M. Parker J. C. (2009). Pulmonary vascular heterogeneity and the starling hypothesis. *Microvas. Res.* 78 71–77. 10.1016/j.mvr.2009.03.004 19332080

[B43] Emr B. Gatto L. Roy S. Satalin J. Ghosh A. Snyder K. (2013). Airway pressure release ventilation prevents ventilator-induced lung injury in normal lungs. *JAMA Surg.* 148 1005–1012. 10.1001/jamasurg.2013.3746 24026214

[B44] Escolar J. D. Escolar A. (2004). Lung hysteresis: a morphological view. *Histol. Histopathol.* 19 159–166. 10.14670/HH-19.159 14702184

[B45] Fan E. Brodie D. Slutsky A. S. (2018). Acute respiratory distress syndrome: advances in diagnosis and treatment. *JAMA* 319 698–710. 29466596 10.1001/jama.2017.21907

[B46] Fan E. Del Sorbo L. Goligher E. C. Hodgson C. L. Munshi L. Walkey A. J. (2017). An official American Thoracic society/European society of intensive care medicine/society of critical care medicine clinical Practice Guideline: mechanical ventilation in adult patients with acute respiratory distress syndrome. *Am. J. Respir. Crit. Care Med.* 195 1253–1263. 10.1164/rccm.201703-0548ST 28459336

[B47] Fan E. Needham D. M. Stewart T. E. (2005). Ventilatory management of acute lung injury and acute respiratory distress syndrome. *JAMA* 294 2889–2896. 16352797 10.1001/jama.294.22.2889

[B48] Fan E. Wilcox M. E. Brower R. G. Stewart T. E. Mehta S. Lapinsky S. E. (2008). Recruitment maneuvers for acute lung injury: a systematic review. *Am. J. Respir. Crit. Care Med.* 178 1156–1163. 10.1164/rccm.200802-335OC 18776154

[B49] Farias L. L. Faffe D. S. Xisto D. G. Santana M. C. Lassance R. Prota L. F. (2005). Positive end-expiratory pressure prevents lung mechanical stress caused by recruitment/derecruitment. *J. Appl. Physiol.* 98 53–61. 10.1152/japplphysiol.00118.2004 15377644

[B50] Faridy E. E. Permutt S. Riley R. L. (1966). Effect of ventilation on surface forces in excised dogs’ lungs. *J. Appl. Physiol.* 21 1453–1462. 10.1152/jappl.1966.21.5.1453 5923215

[B51] Ferguson N. D. Cook D. J. Guyatt G. H. Mehta S. Hand L. Austin P. (2013). High-frequency oscillation in early acute respiratory distress syndrome. *N. Engl. J. Med.* 368 795–805. 10.1056/NEJMoa1215554 23339639

[B52] Gattinoni L. Caironi P. Cressoni M. Chiumello D. Ranieri V. M. Quintel M. (2006). Lung recruitment in patients with the acute respiratory distress syndrome. *N. Engl. J. Med.* 354 1775–1786. 16641394 10.1056/NEJMoa052052

[B53] Gattinoni L. Carlesso E. Caironi P. (2012). Stress and strain within the lung. *Curr. Opin. Crit. Care* 18 42–47. 10.1097/mcc.0b013e32834f17d9 22157254

[B54] Gattinoni L. Collino F. Maiolo G. Rapetti F. Romitti F. Tonetti T. (2017). Positive end-expiratory pressure: how to set it at the individual level. *Ann. Transl. Med.* 5:288. 10.21037/atm.2017.06.64 28828363 PMC5537121

[B55] Gattinoni L. Pelosi P. Crotti S. Valenza F. (1995). Effects of positive end-expiratory pressure on regional distribution of tidal volume and recruitment in adult respiratory distress syndrome. *Am. J. Respir. Crit. Care Med.* 151 1807–1814. 10.1164/ajrccm.151.6.7767524 7767524

[B56] Gattinoni L. Pesenti A. (2005). The concept of “baby lung”. *Intensive Care Med.* 31 776–784. 10.1007/s00134-005-2627-z 15812622

[B57] Gattinoni L. Vagginelli F. Carlesso E. Taccone P. Conte V. Chiumello D. (2003). Decrease in PaCO2 with prone position is predictive of improved outcome in acute respiratory distress syndrome. *Crit. Care Med.* 31 2727–2733. 10.1097/01.ccm.0000098032.34052.f9 14668608

[B58] Ghadiali S. N. Gaver D. P. (2008). Biomechanics of liquid-epithelium interactions in pulmonary airways. *Respir. Physiol. Neurobiol.* 163 232–243. 10.1016/j.resp.2008.04.008 18511356 PMC2652855

[B59] Goligher E. C. Hodgson C. L. Adhikari N. K. J. Meade M. O. Wunsch H. Uleryk E. (2017). Lung recruitment maneuvers for adult patients with acute respiratory distress syndrome. a systematic review and meta-analysis. *Ann. Am. Thorac. Soc.* 14(Suppl. 4) S304–S311. 29043837 10.1513/AnnalsATS.201704-340OT

[B60] Grune J. Tabuchi A. Kuebler W. M. (2019). Alveolar dynamics during mechanical ventilation in the healthy and injured lung. *Intensive Care Med. Exp.* 7(Suppl. 1):34. 10.1186/s40635-019-0226-5 31346797 PMC6658629

[B61] Guerin C. Reignier J. Richard J. C. (2013). Prone positioning in the acute respiratory distress syndrome. *N. Engl. J. Med.* 369 980–981. 10.1056/NEJMc1308895 24004127

[B62] Guldner A. Braune A. Ball L. Silva P. L. Samary C. Insorsi A. (2016). Comparative effects of volutrauma and atelectrauma on lung inflammation in experimental acute respiratory distress syndrome. *Crit. Care Med.* 44 e854–e865. 10.1097/CCM.0000000000001721 27035236 PMC5105831

[B63] Habashi N. M. (2005). Other approaches to open-lung ventilation: airway pressure release ventilation. *Crit. Care Med.* 33(Suppl. 3) S228–S240. 15753733 10.1097/01.ccm.0000155920.11893.37

[B64] Halter J. M. Steinberg J. M. Schiller H. J. DaSilva M. Gatto L. A. Landas S. (2003). Positive end-expiratory pressure after a recruitment maneuver prevents both alveolar collapse and recruitment/derecruitment. *Am. J. Respir. Crit. Care Med.* 167 1620–1626. 10.1164/rccm.200205-435oc 12615628

[B65] Hamlington K. L. Bates J. H. T. Roy G. S. Julianelle A. J. Charlebois C. Suki B. (2018). Alveolar leak develops by a rich-get-richer process in ventilator-induced lung injury. *PLoS One* 13:e0193934. 10.1371/journal.pone.0193934 29590136 PMC5874026

[B66] Hamlington K. L. Ma B. Smith B. J. Bates J. H. (2016). Modeling the progression of epithelial leak caused by overdistension. *Cell. Mol. Bioeng.* 9 151–161. 10.1007/s12195-015-0426-3 26951764 PMC4778393

[B67] Hirshberg E. L. Lanspa M. J. Peterson J. Carpenter L. Wilson E. L. Brown S. M. (2018). Randomized feasibility trial of a low tidal volume-airway pressure release ventilation protocol compared with traditional airway pressure release ventilation and volume control ventilation protocols. *Crit. Care Med.* 46 1943–1952. 10.1097/CCM.0000000000003437 30277890 PMC6250244

[B68] Hodgson C. L. Cooper D. J. Arabi Y. King V. Bersten A. Bihari S. (2019). Maximal Recruitment open lung ventilation in acute respiratory distress syndrome (PHARLAP): a phase II, multicenter, randomized, controlled trial. *Am. J. Respir. Crit. Care Med.* 200 1363–1372. 10.1164/rccm.201901-0109OC 31356105

[B69] Huynh T. T. Liesching T. N. Cereda M. Lei Y. Frazer M. J. Nahouraii M. R. (2019). Efficacy of oscillation and lung expansion in reducing postoperative pulmonary complication. *J. Am. Coll. Surg.* 229 458.e–466.e. 10.1016/j.jamcollsurg.2019.06.004 31362061

[B70] Jain S. V. Kollisch-Singule M. Sadowitz B. Dombert L. Satalin J. Andrews P. (2016). The 30-year evolution of airway pressure release ventilation (APRV). *Intensive Care Med. Exp.* 4:11. 10.1186/s40635-016-0085-2 27207149 PMC4875584

[B71] Jain S. V. Kollisch-Singule M. Satalin J. Searles Q. Dombert L. Abdel-Razek O. (2017). The role of high airway pressure and dynamic strain on ventilator-induced lung injury in a heterogeneous acute lung injury model. *Intensive Care Med. Exp.* 5:25. 10.1186/s40635-017-0138-1 28497420 PMC5427060

[B72] Kang H. Yang H. Tong Z. (2019). Recruitment manoeuvres for adults with acute respiratory distress syndrome receiving mechanical ventilation: a systematic review and meta-analysis. *J. Crit. Care* 50 1–10. 10.1016/j.jcrc.2018.10.033 30453220 PMC10013696

[B73] Kollisch-Singule M. Andrews P. Satalin J. Gatto L. A. Nieman G. F. Habashi N. M. (2019). The time-controlled adaptive ventilation protocol: mechanistic approach to reducing ventilator-induced lung injury. *Eur. Respir. Rev.* 28:180126. 10.1183/16000617.0126-2018 30996041 PMC9488504

[B74] Kollisch-Singule M. Emr B. Jain S. V. Andrews P. Satalin J. Liu J. (2015a). The effects of airway pressure release ventilation on respiratory mechanics in extrapulmonary lung injury. *Intensive Care Med. Exp.* 3:35. 10.1186/s40635-015-0071-0 PMC4688284 26694915

[B75] Kollisch-Singule M. Emr B. Smith B. Roy S. Jain S. Satalin J. (2014a). Mechanical breath profile of airway pressure release ventilation: the effect on alveolar recruitment and microstrain in acute lung injury. *JAMA Surg.* 149 1138–1145. 10.1001/jamasurg.2014.1829 25230047

[B76] Kollisch-Singule M. Emr B. Smith B. Ruiz C. Roy S. Meng Q. (2014b). Airway pressure release ventilation reduces conducting airway micro-strain in lung injury. *J. Am. Coll. Surg.* 219 968–976. 10.1016/j.jamcollsurg.2014.09.011 25440027 PMC4350231

[B77] Kollisch-Singule M. Jain S. Andrews P. Smith B. J. Hamlington-Smith K. L. Roy S. (2015b). Effect of airway pressure release ventilation on dynamic alveolar heterogeneity. *JAMA Surg.* 151 64–72. 10.1001/jamasurg.2015.2683 26444302

[B78] Kollisch-Singule M. Jain S. V. Satalin J. Andrews P. Searles Q. Liu Z. (2017). Limiting ventilator-associated lung injury in a preterm porcine neonatal model. *J. Pediatr. Surg.* 52 50–55. 10.1016/j.jpedsurg.2016.10.020 27837992

[B79] Kollisch-Singule M. C. Jain S. V. Andrews P. L. Satalin J. Gatto L. A. Villar J. (2018). Last word on viewpoint: looking beyond macrovenitlatory parameters and rethinking ventilator-induced lung injury. *J. Appl. Physiol.* 124 1220–1221. 10.1152/japplphysiol.00049.2018 29745822

[B80] Laffey J. G. Bellani G. Pham T. Fan E. Madotto F. Bajwa E. K. (2016). Potentially modifiable factors contributing to outcome from acute respiratory distress syndrome: the LUNG SAFE study. *Intensive Care Med.* 42 1865–1876. 10.1007/s00134-016-4571-5 27757516

[B81] Lalgudi Ganesan S. Jayashree M. Singhi S. C. Bansal A. (2018). Airway pressure release ventilation in pediatric acute respiratory distress syndrome: a randomized controlled trial. *Am. J. Respir. Crit. Care Med.* 198 1199–1207. 10.1164/rccm.201705-0989oc 29641221

[B82] Lewis J. F. Ikegami M. Jobe A. H. Absolom D. (1993). Physiologic responses and distribution of aerosolized surfactant (Survanta) in a nonuniform pattern of lung injury. *Am. Rev. Respir. Dis.* 147(6 Pt 1) 1364–1370. 10.1164/ajrccm/147.6_pt_1.1364 8503547

[B83] Li Bassi G. Marti J. D. Comaru T. Aguilera-Xiol E. Rigol M. Ntoumenopoulos G. (2019). Short-term appraisal of the effects and safety of manual versus ventilator hyperinflation in an animal model of severe pneumonia. *Respir. Care* 64 760–770. 10.4187/respcare.06487 31088989

[B84] Lim J. Litton E. (2019). Airway pressure release ventilation in adult patients with acute hypoxemic respiratory failure: a systematic review and meta-analysis. *Crit. Care Med.* 47 1794–1799. 10.1097/ccm.0000000000003972 31517696

[B85] Lu J. Wang X. Chen M. Cheng L. Chen Q. Jiang H. (2017). An open lung strategy in the management of acute respiratory distress syndrome: a systematic review and meta-analysis. *Shock* 48 43–53. 10.1097/shk.0000000000000822 28125527

[B86] Lutz D. Gazdhar A. Lopez-Rodriguez E. Ruppert C. Mahavadi P. Gunther A. (2015). Alveolar derecruitment and collapse induration as crucial mechanisms in lung injury and fibrosis. *Am. J. Respir. Cell Mol. Biol.* 52 232–243. 10.1165/rcmb.2014-0078OC 25033427

[B87] Maca J. Jor O. Holub M. Sklienka P. Bursa F. Burda M. (2017). Past and present ARDS mortality rates: a systematic review. *Respir. Care* 62 113–122. 10.4187/respcare.04716 27803355

[B88] Magalhaes P. A. F. Padilha G. A. Moraes L. Santos C. L. Maia L. A. Braga C. L. (2018). Effects of pressure support ventilation on ventilator-induced lung injury in mild acute respiratory distress syndrome depend on level of positive end-expiratory pressure: a randomised animal study. *Eur. J. Anaesthesiol.* 35 298–306. 10.1097/EJA.0000000000000763 29324568

[B89] Mahajan M. DiStefano D. Satalin J. Andrews P. Al-Khalisy H. Baker S. (2019). Time-controlled adaptive ventilation (TCAV) accelerates simulated mucus clearance via increased expiratory flow rate. *Intensive Care Med. Exp.* 7:27. 10.1186/s40635-019-0250-5 31098761 PMC6522588

[B90] Majumdar A. Arold S. P. Bartolak-Suki E. Parameswaran H. Suki B. (2012). Jamming dynamics of stretch-induced surfactant release by alveolar type II cells. *J. Appl. Physiol.* 112 824–831. 10.1152/japplphysiol.00975.2010 22033531 PMC3311662

[B91] Makiyama A. M. Gibson L. J. Harris R. S. Venegas J. G. (2014). Stress concentration around an atelectatic region: a finite element model. *Respir. Physiol. Neurobiol.* 201 101–110. 10.1016/j.resp.2014.06.017 25048678

[B92] Manning H. L. Mahler D. A. (2001). Pathophysiology of dyspnea. *Monaldi Arch. Chest Dis.* 56 325–330. 11770215

[B93] Marini J. J. (2018). Dissipation of energy during the respiratory cycle: conditional importance of ergotrauma to structural lung damage. *Curr. Opin. Crit. Care* 24 16–22. 10.1097/MCC.0000000000000470 29176330

[B94] Martin G. S. Brigham K. L. (2012). Fluid flux and clearance in acute lung injury. *Compr. Physiol.* 2 2471–2480. 10.1002/cphy.c100050 23720254

[B95] McNicholas B. A. Rooney G. M. Laffey J. G. (2018). Lessons to learn from epidemiologic studies in ARDS. *Curr. Opin. Crit. Care* 24 41–48. 10.1097/MCC.0000000000000473 29135617

[B96] Mead J. Takishima T. Leith D. (1970). Stress distribution in lungs: a model of pulmonary elasticity. *J. Appl. Physiol.* 28 596–608. 10.1152/jappl.1970.28.5.596 5442255

[B97] Meade M. O. Cook D. J. Guyatt G. H. Slutsky A. S. Arabi Y. M. Cooper D. J. (2008). Ventilation strategy using low tidal volumes, recruitment maneuvers, and high positive end-expiratory pressure for acute lung injury and acute respiratory distress syndrome: a randomized controlled trial. *JAMA* 299 637–645. 18270352 10.1001/jama.299.6.637

[B98] Mellott K. G. Grap M. J. Munro C. L. Sessler C. N. Wetzel P. A. (2009). Patient-ventilator dyssynchrony: clinical significance and implications for practice. *Crit. Care Nurse* 29 41–55 quiz 1 p following 55. 19724065 10.4037/ccn2009612 PMC3742330

[B99] Mercat A. Richard J. C. Vielle B. Jaber S. Osman D. Diehl J. L. (2008). Positive end-expiratory pressure setting in adults with acute lung injury and acute respiratory distress syndrome: a randomized controlled trial. *JAMA* 299 646–655. 18270353 10.1001/jama.299.6.646

[B100] Morais C. C. A. Koyama Y. Yoshida T. Plens G. M. Gomes S. Lima C. A. S. (2018). High positive end-expiratory pressure renders spontaneous effort noninjurious. *Am. J. Respir. Crit. Care Med.* 197 1285–1296. 10.1164/rccm.201706-1244OC 29323536 PMC5955057

[B101] Motta-Ribeiro G. C. Hashimoto S. Winkler T. Baron R. M. Grogg K. Paula L. (2018). Deterioration of regional lung strain and inflammation during early lung injury. *Am. J. Respir. Crit. Care Med.* 198 891–902. 10.1164/rccm.201710-2038OC 29787304 PMC6173064

[B102] Muscedere J. G. Mullen J. B. Gan K. Slutsky A. S. (1994). Tidal ventilation at low airway pressures can augment lung injury. *Am. J. Respir. Crit. Care Med.* 149 1327–1334. 10.1164/ajrccm.149.5.8173774 8173774

[B103] Nakazawa K. Yokoyama K. Yamakawa N. Makita K. (2007). Effect of positive end-expiratory pressure on inflammatory response in oleic acid-induced lung injury and whole-lung lavage-induced lung injury. *J. Anesth.* 21 47–54. 10.1007/s00540-006-0465-y 17285413

[B104] Neumann P. Berglund J. E. Andersson L. G. Maripu E. Magnusson A. Hedenstierna G. (2000). Effects of inverse ratio ventilation and positive end-expiratory pressure in oleic acid-induced lung injury. *Am. J. Respir. Crit. Care Med.* 161 1537–1545. 10.1164/ajrccm.161.5.9906060 10806151

[B105] Neumann P. Berglund J. E. Fernandez Mondejar E. Magnusson A. Hedenstierna G. (1998a). Dynamics of lung collapse and recruitment during prolonged breathing in porcine lung injury. *J. Appl. Physiol.* 85 1533–1543. 10.1152/jappl.1998.85.4.1533 9760351

[B106] Neumann P. Berglund J. E. Mondejar E. F. Magnusson A. Hedenstierna G. (1998b). Effect of different pressure levels on the dynamics of lung collapse and recruitment in oleic-acid-induced lung injury. *Am. J. Respir. Crit. Care Med.* 158(5 Pt 1) 1636–1643. 10.1164/ajrccm.158.5.9711095 9817719

[B107] Nieman G. Gatto L. Andrews P. Satalin J. Camporota L. Daxon B. (2019). Prevention and treatment of acute lung injury with time controlled adaptive ventilation. *Ann. Intensive Care* 10:3. 10.1186/s13613-019-0619-3 PMC6944723 31907704

[B108] Nieman G. F. Andrews P. Satalin J. Wilcox K. Kollisch-Singule M. Madden M. (2018). Acute lung injury: how to stabilize a broken lung. *Critical Care* 22:136. 10.1186/s13054-018-2051-8 29793554 PMC5968707

[B109] Nieman G. F. Bredenberg C. E. (1985). High surface tension pulmonary edema induced by detergent aerosol. *J. Appl. Physiol.* 58 129–136. 10.1152/jappl.1985.58.1.129 2578444

[B110] Nieman G. F. Gatto L. A. Habashi N. M. (2015). Impact of mechanical ventilation on the pathophysiology of progressive acute lung injury. *J. Appl. Physiol.* 119 1245–1261. 10.1152/japplphysiol.00659.2015 26472873

[B111] Nieman G. F. Satalin J. Andrews P. Aiash H. Habashi N. M. Gatto L. A. (2017a). Personalizing mechanical ventilation according to physiologic parameters to stabilize alveoli and minimize ventilator induced lung injury (VILI). *Intensive Care Med. Exp.* 5:8. 10.1186/s40635-017-0121-x 28150228 PMC5289131

[B112] Nieman G. F. Satalin J. Kollisch-Singule M. Andrews P. Aiash H. Habashi N. M. (2017b). Physiology in medicine: understanding dynamic alveolar physiology to minimize ventilator-induced lung injury. *J. Appl. Physiol.* 122 1516–1522. 10.1152/japplphysiol.00123.2017 28385915 PMC7203565

[B113] Papazian L. Aubron C. Brochard L. Chiche J. D. Combes A. Dreyfuss D. (2019). Formal guidelines: management of acute respiratory distress syndrome. *Ann. Intensive Care* 9:69. 10.1186/s13613-019-0540-9 31197492 PMC6565761

[B114] Pavone L. A. Albert S. Carney D. Gatto L. A. Halter J. M. Nieman G. F. (2007). Injurious mechanical ventilation in the normal lung causes a progressive pathologic change in dynamic alveolar mechanics. *Critical Care* 11:R64. 17565688 10.1186/cc5940 PMC2206429

[B115] Perlman C. E. Lederer D. J. Bhattacharya J. (2011). Micromechanics of alveolar edema. *Am. J. Respir. Cell Mol. Biol.* 44 34–39. 10.1165/rcmb.2009-0005OC 20118224 PMC3028256

[B116] Perun M. L. Gaver D. P. (1995). Interaction between airway lining fluid forces and parenchymal tethering during pulmonary airway reopening. *J. Appl. Physiol.* 79 1717–1728. 10.1152/jappl.1995.79.5.1717 8594034

[B117] Petrucci N. De Feo C. (2013). Lung protective ventilation strategy for the acute respiratory distress syndrome. *Cochrane Database Syst. Rev.* 2013:CD003844. 10.1002/14651858.CD003844.pub4 PMC6517299 23450544

[B118] Pham T. Serpa Neto A. Pelosi P. Laffey J. G. De Haro C. Lorente J. A. (2019). Outcomes of patients presenting with mild acute respiratory distress syndrome: insights from the LUNG SAFE study. *Anesthesiology* 130 263–283. 10.1097/ALN.0000000000002508 30499850

[B119] Phua J. Badia J. R. Adhikari N. K. Friedrich J. O. Fowler R. A. Singh J. M. (2009). Has mortality from acute respiratory distress syndrome decreased over time: a systematic review. *Am. J. Respir. Crit. Care Med.* 179 220–227. 10.1164/rccm.200805-722OC 19011152

[B120] Protti A. Andreis D. T. Iapichino G. E. Monti M. Comini B. Milesi M. (2013a). High positive end-expiratory pressure: only a dam against oedema formation? *Crit. Care* 17:R131. 10.1186/cc12810 23844622 PMC4056428

[B121] Protti A. Andreis D. T. Monti M. Santini A. Sparacino C. C. Langer T. (2013b). Lung stress and strain during mechanical ventilation: any difference between statics and dynamics? *Crit. Care Med.* 41 1046–1055. 10.1097/CCM.0b013e31827417a6 23385096

[B122] Protti A. Votta E. Gattinoni L. (2014). Which is the most important strain in the pathogenesis of ventilator-induced lung injury: dynamic or static? *Curr. Opin. Crit. Care* 20 33–38. 10.1097/MCC.0000000000000047 24247615

[B123] Putensen C. Theuerkauf N. Zinserling J. Wrigge H. Pelosi P. (2009). Meta-analysis: ventilation strategies and outcomes of the acute respiratory distress syndrome and acute lung injury. *Ann. Intern. Med.* 151 566–576. 19841457 10.7326/0003-4819-151-8-200910200-00011

[B124] Rahaman U. (2017). Mathematics of ventilator-induced lung injury. *Indian J. Crit. Care Med.* 21 521–524. 28904482 10.4103/ijccm.IJCCM_411_16 PMC5588487

[B125] Raymondos K. Dirks T. Quintel M. Molitoris U. Ahrens J. Dieck T. (2017). Outcome of acute respiratory distress syndrome in university and non-university hospitals in Germany. *Crit. Care* 21:122. 10.1186/s13054-017-1687-0 28554331 PMC5448143

[B126] Retamal J. Bergamini B. C. Carvalho A. R. Bozza F. A. Borzone G. Borges J. B. (2014). Non-lobar atelectasis generates inflammation and structural alveolar injury in the surrounding healthy tissue during mechanical ventilation. *Critical Care* 18:505. 10.1186/s13054-014-0505-1 25200702 PMC4172813

[B127] Rezoagli E. Fumagalli R. Bellani G. (2017). Definition and epidemiology of acute respiratory distress syndrome. *Ann Transl Med.* 5:282. 10.21037/atm.2017.06.62 28828357 PMC5537110

[B128] Roy S. Habashi N. Sadowitz B. Andrews P. Ge L. Wang G. (2013). Early airway pressure release ventilation prevents Ards-a novel preventive approach to lung injury. *Shock* 39 28–38. 10.1097/SHK.0b013e31827b47bb 23247119 PMC3539171

[B129] Roy S. Sadowitz B. Andrews P. Gatto L. A. Marx W. Ge L. (2012). Early stabilizing alveolar ventilation prevents acute respiratory distress syndrome: a novel timing-based ventilatory intervention to avert lung injury. *J. Trauma Acute Care Surg.* 73 391–400. 10.1097/ta.0b013e31825c7a82 22846945 PMC3521044

[B130] Roy S. K. Emr B. Sadowitz B. Gatto L. A. Ghosh A. Satalin J. M. (2013). Preemptive application of airway pressure release ventilation prevents development of acute respiratory distress syndrome in a rat traumatic hemorrhagic shock model. *Shock* 40 210–216. 10.1097/SHK.0b013e31829efb06 23799354 PMC3780366

[B131] Ruhl N. Lopez-Rodriguez E. Albert K. Smith B. J. Weaver T. E. Ochs M. (2019). Surfactant protein B deficiency induced high surface tension: relationship between alveolar micromechanics, alveolar fluid properties and alveolar epithelial cell injury. *Int. J. Mol. Sci.* 20:4243. 10.3390/ijms20174243 31480246 PMC6747270

[B132] Saddy F. Moraes L. Santos C. L. Oliveira G. P. Cruz F. F. Morales M. M. (2013). Biphasic positive airway pressure minimizes biological impact on lung tissue in mild acute lung injury independent of etiology. *Crit. Care* 17:R228. 10.1186/cc13051 24103805 PMC4057608

[B133] Sahetya S. K. Brower R. G. (2017). Lung recruitment and titrated PEEP in moderate to severe ARDS: is the door closing on the open lung? *JAMA* 318 1327–1329. 28973075 10.1001/jama.2017.13695 PMC7297277

[B134] Sahetya S. K. Mancebo J. Brower R. G. (2017). Fifty years of research in ARDS. Vt selection in acute respiratory distress syndrome. *Am. J. Respir. Crit. Care Med.* 196 1519–1525. 10.1164/rccm.201708-1629CI 28930639 PMC5754449

[B135] Satalin J. Habashi N. M. Nieman G. F. (2018). Never give the lung the opportunity to collapse. *Trends Anaesth. Crit. Care* 22 10–16. 10.1016/j.tacc.2018.05.007

[B136] Schiller H. J. McCann U. G. Carney D. E. Gatto L. A. Steinberg J. M. Nieman G. F. (2001). Altered alveolar mechanics in the acutely injured lung. *Crit. Care Med.* 29 1049–1055. 10.1097/00003246-200105000-00036 11383531

[B137] Seah A. S. Grant K. A. Aliyeva M. Allen G. B. Bates J. H. T. (2011). Quantifying the roles of tidal volume and PEEP in the pathogenesis of ventilator-induced lung injury. *Ann. Biomed. Eng.* 39 1505–1516. 10.1007/s10439-010-0237-6 21203845

[B138] Shen Y. Cai G. Gong S. Dong L. Yan J. Cai W. (2019). Interaction between low tidal volume ventilation strategy and severity of acute respiratory distress syndrome: a retrospective cohort study. *Critical Care* 23:254. 10.1186/s13054-019-2530-6 31300012 PMC6626332

[B139] Silva P. L. Cruz F. F. Samary C. D. S. Moraes L. de Magalhaes R. F. Fernandes M. V. S. (2018). Biological response to time-controlled adaptive ventilation depends on acute respiratory distress syndrome etiology. *Critical Care Med.* 46 e609–e617. 10.1097/CCM.0000000000003078 29485489

[B140] Slutsky A. S. Ranieri V. M. (2013). Ventilator-induced lung injury. *N. Engl. J. Med.* 369 2126–2136. 24283226 10.1056/NEJMra1208707

[B141] Smith B. J. Lundblad L. K. Kollisch-Singule M. Satalin J. Nieman G. Habashi N. (2015). Predicting the response of the injured lung to the mechanical breath profile. *J. Appl. Physiol.* 118 932–940. 10.1152/japplphysiol.00902.2014 25635004 PMC4385881

[B142] Solomon I. C. Edelman N. H. Neubauer J. A. (2000). Pre-Botzinger complex functions as a central hypoxia chemosensor for respiration in vivo. *J. Neurophysiol.* 83 2854–2868. 10.1152/jn.2000.83.5.2854 10805683

[B143] Stewart T. E. Meade M. O. Cook D. J. Granton J. T. Hodder R. V. Lapinsky S. E. (1998). Evaluation of a ventilation strategy to prevent barotrauma in patients at high risk for acute respiratory distress syndrome. Pressure- and volume-limited ventilation strategy group. *N. Engl. J. Med.* 338 355–361. 10.1056/nejm199802053380603 9449728

[B144] Suki B. Barabasi A. L. Hantos Z. Petak F. Stanley H. E. (1994). Avalanches and power-law behaviour in lung inflation. *Nature* 368 615–618. 10.1038/368615a0 8145846

[B145] Suki B. Bates J. H. (2011). Emergent behavior in lung structure and function. *J. Appl. Physiol.* 110 1109–1110. 10.1152/japplphysiol.00179.2011 21310887

[B146] Taeusch H. W. Bernardino de la Serna J. Perez-Gil J. Alonso C. Zasadzinski J. A. (2005). Inactivation of pulmonary surfactant due to serum-inhibited adsorption and reversal by hydrophilic polymers: experimental. *Biophys. J.* 89 1769–1779. 10.1529/biophysj.105.062620 15923228 PMC1366680

[B147] Takahashi A. Bartolak-Suki E. Majumdar A. Suki B. (2015). Changes in respiratory elastance after deep inspirations reflect surface film functionality in mice with acute lung injury. *J. Appl. Physiol.* 119 258–265. 10.1152/japplphysiol.00476.2014 26066828 PMC4526701

[B148] Talab H. F. Zabani I. A. Abdelrahman H. S. Bukhari W. L. Mamoun I. Ashour M. A. (2009). Intraoperative ventilatory strategies for prevention of pulmonary atelectasis in obese patients undergoing laparoscopic bariatric surgery. *Anesth. Analg.* 109 1511–1516. 10.1213/ANE.0b013e3181ba7945 19843790

[B149] Thompson B. T. Chambers R. C. Liu K. D. (2017). Acute respiratory distress syndrome. *N. Engl. J. Med.* 377 1904–1905. 10.1056/NEJMc1711824 29117492

[B150] Tojo K. Yoshida T. Yazawa T. Goto T. (2018). Driving-pressure-independent protective effects of open lung approach against experimental acute respiratory distress syndrome. *Crit. Care* 22:228. 10.1186/s13054-018-2154-2 30243301 PMC6151188

[B151] Tonetti T. Cressoni M. Collino F. Maiolo G. Rapetti F. Quintel M. (2017). Volutrauma, atelectrauma, and mechanical power. *Crit. Care Med.* 45 e327–e328. 10.1097/ccm.0000000000002193 28212229

[B152] van der Zee P. Gommers D. (2019). Recruitment maneuvers and higher PEEP, the so-called open lung concept, in patients with ARDS. *Crit. Care* 23:73. 10.1186/s13054-019-2365-1 30850004 PMC6408810

[B153] Villar J. Blanco J. Anon J. M. Santos-Bouza A. Blanch L. Ambros A. (2011). The ALIEN study: incidence and outcome of acute respiratory distress syndrome in the era of lung protective ventilation. *Intensive Care Med.* 37 1932–1941. 10.1007/s00134-011-2380-4 21997128

[B154] Villar J. Blanco J. Kacmarek R. M. (2016). Current incidence and outcome of the acute respiratory distress syndrome. *Curr. Opin. Crit. Care.* 22 1–6. 10.1097/MCC.0000000000000266 26645551

[B155] Webb H. H. Tierney D. F. (1974). Experimental pulmonary edema due to intermittent positive pressure ventilation with high inflation pressures. Protection by positive end-expiratory pressure. *Am. Rev. Respir. Dis.* 110 556–565. 4611290 10.1164/arrd.1974.110.5.556

[B156] Wellman T. J. de Prost N. Tucci M. Winkler T. Baron R. M. Filipczak P. (2016). Lung metabolic activation as an early biomarker of acute respiratory distress syndrome and local gene expression heterogeneity. *Anesthesiology* 125 992–1004. 10.1097/aln.0000000000001334 27611185 PMC5096592

[B157] Wellman T. J. Winkler T. Costa E. L. Musch G. Harris R. S. Zheng H. (2014). Effect of local tidal lung strain on inflammation in normal and lipopolysaccharide-exposed sheep. *Crit. Care Med.* 42 e491–e500. 10.1097/CCM.0000000000000346 24758890 PMC4123638

[B158] Widdicombe J. (2001). Airway receptors. *Respir. Physiol.* 125 3–15. 11240149 10.1016/s0034-5687(00)00201-2

[B159] Wirtz H. R. Dobbs L. G. (1990). Calcium mobilization and exocytosis after one mechanical stretch of lung epithelial cells. *Science* 250 1266–1269. 10.1126/science.2173861 2173861

[B160] Xin Y. Cereda M. Hamedani H. Pourfathi M. Siddiqui S. Meeder N. (2018). Unstable inflation causing injury. Insight from prone position and paired computed tomography scans. *Am. J. Respir. Crit. Care Med.* 198 197–207. 10.1164/rccm.201708-1728OC 29420904 PMC6058981

[B161] Yoshida T. Fujino Y. Amato M. B. Kavanagh B. P. (2017). nisms, and management. *Am. J. Respir. Crit. Care Med.* 195 985–992. 10.1164/rccm.201604-0748CP 27786562

[B162] Young D. Lamb S. E. Shah S. MacKenzie I. Tunnicliffe W. Lall R. (2013). High-frequency oscillation for acute respiratory distress syndrome. *N. Engl. J. Med.* 368 806–813. 10.1056/NEJMoa1215716 23339638

[B163] Yu J. (2016). Deflation-activated receptors, not classical inflation-activated receptors, mediate the Hering-Breuer deflation reflex. *J. Appl. Physiol.* 121 1041–1046. 10.1152/japplphysiol.00903.2015 27586839

[B164] Zheng X. Jiang Y. Jia H. Ma W. Han Y. Li W. (2019). Effect of lung recruitment and titrated positive end-expiratory pressure (PEEP) versus low PEEP on patients with moderate-severe acute respiratory distress syndrome: a systematic review and meta-analysis of randomized controlled trials. *Ther. Adv. Respir. Dis.* 13:1753466619858228. 10.1177/1753466619858228 31269867 PMC6611025

[B165] Zhou Y. Jin X. Lv Y. Wang P. Yang Y. Liang G. (2017). Early application of airway pressure release ventilation may reduce the duration of mechanical ventilation in acute respiratory distress syndrome. *Intensive Care Med.* 43 1648–1659. 10.1007/s00134-017-4912-z 28936695 PMC5633625

